# Two‐Step Synthesis of a Chiral Fluorinated Alcohol With Silica‐Supported Enzyme *Rr*ADH in Batch and Continuous Flow Mode

**DOI:** 10.1002/chem.202503304

**Published:** 2026-02-09

**Authors:** Egzon Cermjani, Greta Nölke, Stefano Di Fiore, Christoph Deckers, Doris Hanselmann, Bettina Herbig, Susanne Wintzheimer, Thomas H. Rehm

**Affiliations:** ^1^ Fraunhofer Institute for Microengineering and Microsystems IMM Mainz Germany; ^2^ Department of Chemistry Johannes Gutenberg‐University Mainz Mainz Germany; ^3^ Fraunhofer Institute for Molecular Biology and Applied Ecology IME Aachen Germany; ^4^ Fraunhofer Institute for Silicate Research ISC Würzburg Germany

## Abstract

The growing demand for sustainable and efficient methods for synthesizing fine chemicals has increased interest in innovative approaches for accessing high‐quality chiral building blocks, particularly fluoroalcohols, which are relevant for the production of active pharmaceutical ingredients (APIs). This study presents the complete integration of a two‐step process in a continuous flow reactor system for the synthesis of (*R*)‐2‐fluoro‐1‐phenylethanol as a reference molecule. To this end, the individual reaction steps and technologies for the decarboxylative fluorination of 3‐oxo‐3‐phenylpropanoic acid in aqueous media, followed by an enantioselective biocatalytic reduction of the prochiral intermediate phenacyl fluoride, were adapted and implemented in a compact laboratory system for performance demonstration. Alcohol dehydrogenase (ADH) from *Rhodococcus ruber* (*Rr*ADH) produced in a plant‐derived BY2 cell‐free expression system was used as the biocatalyst, which was immobilized via an imine bond on glutaraldehyde‐modified silica supraparticles. The immobilized enzymes were used in batch mode for comprehensive kinetic studies of the enantioselective reduction, including evaluations of their operational and storage stability. Excellent enantiomeric excess (> 99.9%) and overall yields of up to 91% were achieved for both synthesis steps. These results are a prerequisite for the targeted and stable use of the enzyme in a continuously operated two‐step process, which was achieved by using a serial micro batch reactor (SMBR) setup with a capillary reactor for precise temperature control. This study demonstrates the advantages of combining immobilized biocatalysts with continuous‐flow operation for achieving high efficiency and selectivity in the synthesis of chiral fluoroalcohols. The integrated process provides a sustainable and versatile basis for future developments in the green synthesis of fluorinated building blocks relevant to pharmaceutical applications.

## Graphical abstract

A two‐step process was transferred from batch to flow mode for the synthesis of (*R*)‐2‐fluoro‐1‐phenylethanol as a benchmark, consisting of a decarboxylative fluorination in aqueous media, followed by an enantioselective biocatalytic reduction of the prochiral intermediate with *Rr*ADH immobilized on silica supraparticles. The biocatalyst achieves an excellent enantiomeric excess of >99.9% with an overall yield of 91% for the complete process.

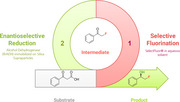

## 1 Introduction

The strategic incorporation of fluorine into biologically active compounds—particularly active pharmaceutical ingredients (APIs)—is a well‐established approach for improving their pharmacokinetic and pharmacodynamic profiles [1]. The significance of this modification is underscored by the high prevalence of fluorinated functional groups in FDA (Food and Drug Administration)‐approved APIs [2, 3]. The development of fast, efficient, and sustainable synthetic routes to enantiopure fluorinated building blocks offers a promising avenue for reducing time and costs, while improving the environmental sustainability associated with API synthesis [4, 5].

Addressing the need for a more sustainable synthesis of fluorinated fine chemicals, continuous flow processing emerges as a powerful approach to enhance efficiency, scalability, and safety. This technology enables automated and continuous chemical production, thereby minimizing the risk of human errors, reducing operational costs, and eliminating unproductive dead times commonly associated with batch manufacturing [6, 7]. It facilitates straightforward process intensification and scale‐up via reactor numbering‐up, often requiring less material investment compared to conventional batch operations [8]. Significant economic and ecological benefits can be realized through the integration of cascade or multi‐step reactions into continuous flow systems, eliminating the need for intermediate isolation, reducing the number of unit operations, and lowering solvent and waste generation [4, 9]. Moreover, meso‐ and microreactors, such as capillary flow reactors or microfluidic chips, are particularly well‐suited for handling complex or sensitive transformations that are often challenging in batch mode [10, 11, 12]. This includes multiphase reactions (e.g, gas–liquid, liquid–liquid, or solid–liquid systems), where continuous flow reactors offer superior phase mixing and mass transfer due to their high surface‐to‐volume ratios. In addition, handling gaseous reagents becomes significantly safer under flow conditions. Enhanced thermal control in small‐volume flow reactors further contributes to improved process safety and reproducibility [12].

The field of sustainable chemistry spans a wide range of disciplines, among which biocatalysis in continuous flow systems has emerged as a particularly promising approach [13, 14]. Biocatalysis, including the use of whole cells, cell lysates, or isolated enzymes, represents a powerful and inherently sustainable strategy, rooted in the catalytic processes of living organisms and widely applied in both laboratory and industrial‐scale synthesis [15, 16]. Enzymes, in particular, enable highly stereoselective transformations under mild reaction conditions with their catalytically active sites. Recent advances in protein engineering, including directed evolution and computational design, have significantly enhanced the discovery, optimization, and functional tailoring of biocatalysts to meet specific synthetic requirements [17, 18, 19]. These developments include both top‐down strategies, which involve the modification of naturally occurring enzymes, and bottom‐up approaches, wherein entirely new catalytic functionalities are introduced into otherwise inactive scaffolds [19]. Biocatalysis already plays a pivotal role in the industrial production of APIs, particularly in stereoselective transformations, where enzymes frequently outperform conventional catalytic methods, such as transition metal catalysis, in terms of selectivity, efficiency, and sustainability [20, 21].

Especially for industrial applications, enzymes must exhibit long‐term operational stability and be economically viable to produce. However, enzyme production is often costly, and their separation or recycling from the reaction medium remains a significant challenge [22]. To address these limitations, particularly in terms of cost‐efficiency [23] and productivity, enzyme immobilization has emerged as a promising strategy. It not only facilitates catalyst reuse but can also enhance enzyme stability sometimes [24, 25, 26].

Immobilization typically involves linking enzymes onto solid supports through various techniques, including physical entrapment within polymeric networks, cross‐linking to support materials, or attachment via ionic or covalent bonding to solid surfaces or reactor walls [27, 28]. These immobilized systems can subsequently be deployed in continuous flow settings, such as packed bed reactors (PBRs), fluidized bed systems [29, 30, 31], or potentially as suspended catalysts. Despite these advantages, enzyme immobilization presents certain challenges. Activity losses upon immobilization are common, and the economic viability of the approach depends heavily on the choice of immobilization method [24, 32]. Consequently, the development and application of efficient and cost‐effective immobilization strategies remains crucial to unlocking the full potential of biocatalysis in industrial continuous flow applications [33].

### 1.1 Selection of Technological Approach and (Bio)Chemical Components

The use of larger particles or beads, commonly employed in stirred tank reactors, often leads to mass transfer limitations and exposes the particles to high mechanical stress [34]. In contrast, smaller particles are preferred for improving access to the catalytic surface, which can enhance both mass transfer and overall catalytic efficiency. An advanced approach for employing catalytically active particles in continuous flow systems is the Serial Micro Batch Reactor (SMBR) concept, which utilizes a highly controllable three‐phase slug flow regime, also referred to as Taylor flow [35]. This flow pattern enables the effective application of heterogeneous catalysts in systems that are unsuitable for traditional packed‐bed or fluidized‐bed reactors [29, 36]. In this setup, an inert gas or immiscible liquid [37] is dosed together with the catalyst suspension, generating a segmented flow that promotes internal circulation by toroidal forces, improving mixing and enhanced catalyst accessibility [38]. Compared to packed‐bed systems, which can suffer from catalyst leaching, deactivation, and high pressure drops [12, 36], the SMBR concept offers several advantages for solid–liquid reactions, including extended residence times, lower energy requirements, and reduced pressure losses [26].

**FIGURE 1:**
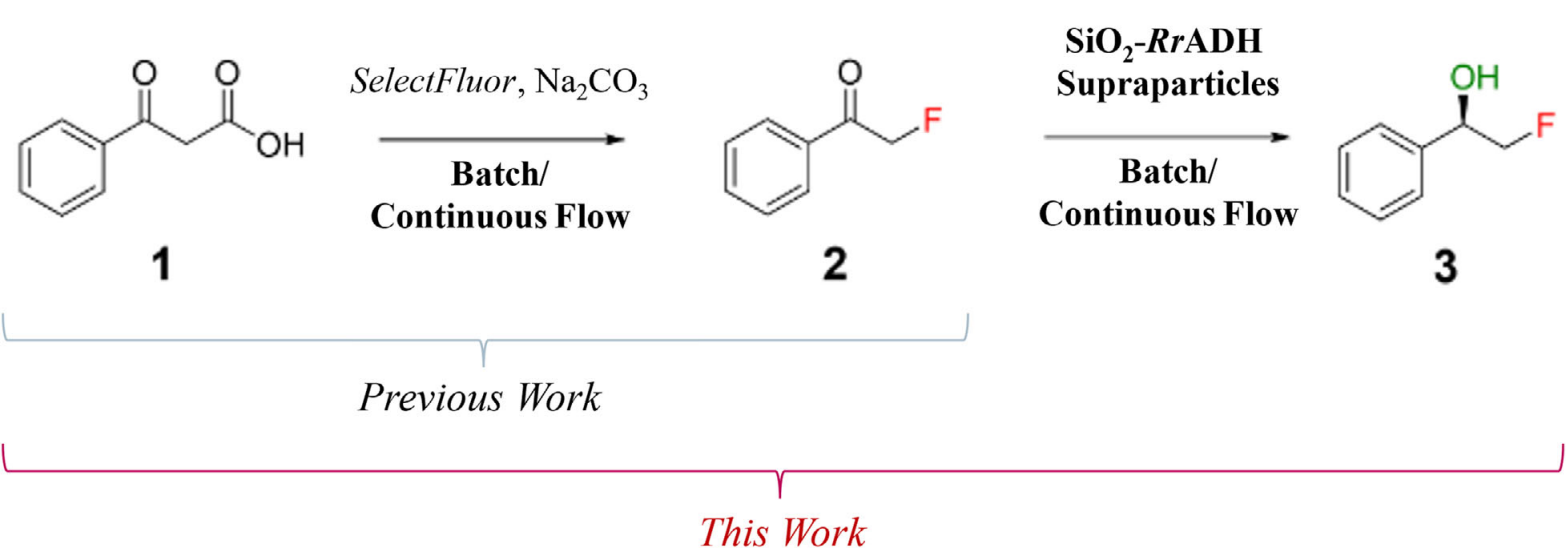
Previous work: Optimized decarboxylative fluorination of 3‐oxo‐3‐phenyl propanoic acid. This Work: Two‐step continuous flow synthesis of (*R*)‐2‐fluoro‐1‐phenyl ethanol, using *Rr*ADH, covalently bound on silica supraparticles. *Rr*ADH, *Rhodococcus ruber*.

Prior research has demonstrated the efficacy of catalyst systems based on so‐called supraparticles [39] for enabling heterogeneous (bio‐)catalysis in continuous flow processes via the use of catalyst suspensions. In these studies, micrometer‐sized porous silica supraparticles, assembled from silica nanoparticles [40] by a spray‐drying process [41], serve as versatile support materials for enzyme immobilization. This assembly method yields highly tunable supraparticles, which can be selectively bio‐ or chemofunctionalized to accommodate specific catalytic functionalities, including enzyme attachment or photocatalyst integration [42]. This modular integration strategy allows for the construction of multifunctional hybrid catalysts capable of conducting complex catalysis. For the technology and performance demonstration, the two‐step synthesis of the API‐relevant fluorinated building block (*R*)‐2‐fluoro‐1‐phenylethanol **3** was selected (Figure 1). This molecule is an ideal candidate as a benchmark substrate for evaluating the synthesis strategy described herein. Owing to its simple molecular structure and its relevance as an API building block, it represents a suitable model compound. The structural motif of a chiral vicinal fluorinated alcohol is present in several APIs, including the fluorinated drug Belzutifan, which is used for the treatment of *Von Hippel–Lindau* disease‐associated tumors [2, 3]. The synthesis of the intermediate phenacyl fluoride **2** is based on a previously described process [43].

For the enantioselective reduction of the intermediate phenacyl fluoride **2**, the alcohol dehydrogenase (ADH)‐A from *Rhodococcus ruber* (*Rr*ADH) [44] is selected, which can catalyze the reversible oxidation/reduction of a variety of alcohols/ketones. *Rr*ADH comprises four monomers with a molecular weight of 38 kDa. Each monomer contains a zinc ion that is catalytically crucial and responsible for the oxidoreductase activity of *Rr*ADH [45, 46]. It is of great interest for its application in the synthesis of chiral fluoro alcohols, as it can facilitate the enantioselective reduction of ketones by catalytic amounts of the cofactor nicotinamide adenine dinucleotide (NAD) and a sacrificial cosubstrate for cofactor regeneration. Asymmetric reductions of substrates with ketoreductases are frequently conducted by a two‐enzyme strategy [47]. As it is well‐documented in the relevant literature, prominent examples of cofactor regeneration include for example glucose dehydrogenase, ADH, and formate dehydrogenase [47, 48]. Despite the documented success of these strategies, their implementation leads to further complications of the respective processes. For pragmatic reasons, the utilization of a singular enzyme system, such as *Rr*ADH, which possesses the capacity to execute the desired reaction and facilitate cofactor regeneration, is favored and selected for this work.

## 2 Materials and Methods

### 2.1 Production of *Rr*ADH in the Plant‐derived BY2 Cell‐Free Expression System

The cDNA fragment encoding ADH from *Rr*ADH [49] was synthesized as a double‐stranded DNA fragment (Integrated DNA Technologies, Coralville, IA, United States). The fragment included an *N*‐terminal StrepII affinity tag sequence and was cloned into the *Nco*I and *Kpn*I sites of the pLenEx vector [50] for in vitro, cell‐free expression under the control of the T7 promoter. The coding sequence was codon‐optimized for *E. coli* using the IDT Codon Optimisation Tool. To facilitate cloning, an additional alanine residue was introduced after the start codon to create an *Nco*I restriction site. Cell‐free synthesis of *Rr*ADH was performed in a single‐pot reaction using 10 mL of tobacco BYL lysate containing all components required for in vitro transcription and translation (T7 polymerase, nucleoside triphosphates, amino acids, and salts), as previously described [51]. The reaction mixture was also supplemented with 40 µM ZnCl_2_, as an essential structural and catalytic cofactor for the ADH activity. Protein expression was carried out for 48 h at 25°C and 70% relative humidity, shaking at 500 rpm, after addition of 10 nM pLENex‐Strep‐RrADH plasmid. Recombinant *Rr*ADH was purified by strep‐tactin affinity chromatography (Strep‐TactinXT 4Flow matrix, IBA) using gravity flow at 4°C according to the manufacturer's instructions. Then, the bound protein was eluted in 2.5 mL of BXT elution buffer (100 mM Tris/HCl, 150 mM NaCl, 50 mM biotin, pH 8). The eluted fraction was equilibrated with 0.1 M potassium phosphate buffer (pH 8) using a Sephadex G‐25 PD10 desalting column (GE Healthcare), aliquoted, and stored at −20°C until use.

### 2.2 Production of Silica Supraparticles With Glutaraldehyde Moieties

SiO_2_ nanoparticles with a diameter of 80 nm were synthesized using a recently published method [40] based on the Stöber process. Briefly, 300 mL of ethanol was combined with 15 mL of a 25 wt% aqueous ammonia solution. The mixture was stirred vigorously while adding 15 g (0.072 mol) of TEOS, and it was then stirred overnight. To purify the nanoparticles, the ethanol/ammonia solution was removed via rotary evaporation, and the mixture was dialyzed for 24 h with multiple water changes using a dialysis membrane (cellulose hydrate membrane, Nadir‐dialysis tubing, Roth, Germany) with a molecular weight cut‐off of 10 kDa. The functionalization of the SiO_2_ nanoparticles with (3‐aminopropyl)triethoxysilane and glutaraldehyde was carried out according to a previously described protocol [52], and the nanoparticles were redispersed in water. For supraparticle fabrication, a dispersion of glutaraldehyde‐functionalized SiO_2_ nanoparticles in water with a solid weight content of 6% was spray‐dried using a B‐290 mini spray‐dryer (Büchi, Switzerland). The spray‐dryer was equipped with a two‐fluid nozzle, the inlet temperature was set to 30°C, and the outlet temperature was approximately 24°C at a flow rate of 1 mL/min.

### 2.3 Immobilization of *Rr*ADH on Silica Supraparticles

Glutaraldehyde‐functionalized SiO_2_ nanoparticles and supraparticles were suspended in 0.1 M potassium phosphate buffer (pH 8). Affinity‐purified *Rr*ADH (1 mg) was added per 100 mg of SiO_2_ particles, and the suspension was gently mixed at 4°C for 12 h using a VWR 360° tube rotator to facilitate enzyme immobilization. Covalent attachment occurred via imine (Schiff base) formation between the aldehyde groups of glutaraldehyde and primary amine groups on lysine residues of *Rr*ADH (Figure S7). Following incubation, ADH‐SiO_2_ particles were collected by centrifugation at 3000 rcf for 3 min and washed three times with cold 0.1 M potassium phosphate buffer (pH 8) to remove unbound protein. The supernatant and wash fractions were collected for the spectrophotometric analysis at 280 nm to assess coupling efficiency. The purified conjugates were stored in the same buffer at −20°C until use.

### 2.4 Characterization Methods

The isoelectric points were determined by titrating with HCl and NaOH and simultaneous zeta‐potential measurements (Zetasizer Nano ZS from Malvern Instruments in combination with the Multi‐Purpose Titrator MPT‐2). For ICP analysis, the samples were digested in 10 mL of 3 weight‐% HNO_3_ for 15 min and then centrifuged at 1500 rcf. The supernatant was retrieved and measured using the Agilent 5800 ICP‐OES Instrument.

## 3 Results and Discussion

### 3.1 Activity of *Rr*ADH for the Reduction of Phenacyl Fluoride 2 to (*R*)‐2‐Fluoro‐1‐Phenylethanol 3

Prior kinetic investigations for *Rr*ADH were performed by Widersten et al. [53] using ketones and secondary alcohols, including acetophenone and (*R*)/(*S*)‐2‐phenylethanol, showing sufficient catalytic activity of *Rr*ADH, demonstrating the enzyme's substantial catalytic activity.

**FIGURE 2:**
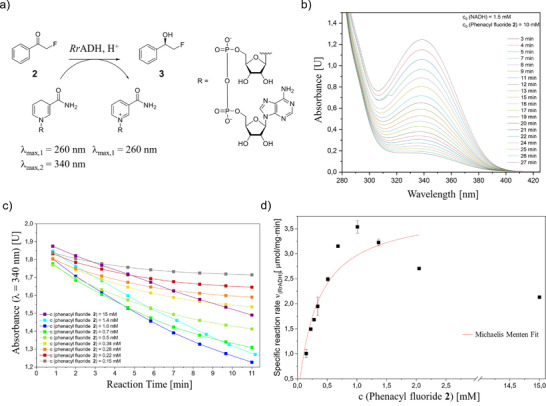
(a) Enantioselective reduction of phenacyl fluoride **2** to (*R*)‐2‐fluoro‐1‐phenylethanol **3**, using *Rr*ADH as catalyst and NADH as reducing agent. NADH shows distinct absorption bands at *λ* = 260 nm (adenine band) and 340 nm (chinoid nicotinamide band). Upon oxidation to NAD^+^, the chinoid structure is lost, diminishing the absorption at *λ* = 340 nm. (b) Time‐dependent decrease in absorbance at *λ* = 340 nm upon reduction of phenacyl fluoride **2** and NADH consumption, catalyzed by *Rr*ADH. (c) Time‐dependent decrease in absorbance at *λ* = 340 nm due to NADH consumption with different starting concentrations of phenacyl fluoride **2**. (d) Specific reaction rates for the reduction of phenacyl fluoride **2** depending on the starting concentration of phenacyl fluoride **2**. In order to determine the initial reaction rates, only the initial linear portions of the reaction progress curves were considered (for further details, see Supporting Information). For the Michaelis‐Menten Fit, data up to c (phenacyl fluoride **3**) = 2.0 mM are used. The error bars represent the standard deviation of two independent experimental datasets (n = 2). *Rr*ADH, *Rhodococcus ruber*.

*Rr*ADH and its performance regarding the enantioselective reduction of phenacyl fluoride **2** to (*R*)‐2‐fluoro‐1‐phenyl ethanol **3** is characterized and evaluated before its immobilization on silica supraparticles as a solid support. In this context, NADH is employed as the reducing agent (Figure 2a). The rate of consumption of NADH by *Rr*ADH is monitored by UV/VIS spectroscopy, by measuring the decrease in light absorption at a wavelength of 340 nm, thus allowing a time‐dependent tracing of the NADH degradation to NAD^+^. Assuming that the reaction kinetics for the respective reaction follow typical Michaelis‐Menten kinetics behavior, it can be deduced that increasing the concentration of phenacyl fluoride **2** or NADH leads to a higher probability that the respective substrate occupies the active center of the enzyme. This, in turn, increases the overall reaction rate and allows the reduction reaction to occur. To ascertain saturating NADH concentrations, experimental rows with NADH concentrations varying from 0.2 to 1.0 mM are performed (Figure S3). The experimental data suggest that no increase in reaction rate is observed upon increasing the initial NADH concentration higher than 0.2 mM.

The calculation of kinetic parameters for the enantioselective reduction of phenacyl fluoride **2** using *Rr*ADH is achieved through the experimental calculation of initial reaction rates. This is accomplished by monitoring the consumption of NADH through UV/VIS spectroscopy, whilst also varying the substrate concentration of phenacyl fluoride **2** (for further details, see Table S4). Subsequent to this, the initial reaction rates of each experimental row are plotted, and the kinetic parameters are calculated by means of a Michaelis‐Menten fit (Figures S4 and S5).

It is evident that by increasing the substrate concentration up to 1 mM at the standard reaction parameters (5 µg/mL *Rr*ADH), higher reaction rates are determined, reaching a maximum reaction rate ν_max_ of approximately 3.89 ± 0.41 µmol/mg•min and a Michaelis‐Menten constant *K_m_* (the substrate concentration, at which half of the maximum reaction rate is reached) of 0.31 mM ± 0.10. Up to a substrate concentration of 1 mM, the kinetics follow typical Michaelis‐Menten kinetics. However, increasing the substrate concentration above 1 mM leads to no further increase in reaction rate, which actually decreases to a reaction rate of 2.7 µmol/mg•min at 2 mM substrate concentration, suggesting that the enzymes are fully occupied with the substrate and indicating inhibitory effects. This decrease in reaction rate might be due to a competitive product inhibition by (*R*)‐2‐fluoro‐1‐phenyl ethanol **3**, induced by its increasing concentration, which is also shown with the acetophenone/1‐phenylethanol system in studies by Widersten et al. [53]. Overall, the *Rr*ADH‐catalyzed reduction of phenacyl fluoride **2** proceeds at high reaction rates, underscoring the enzyme's suitability for this reaction.

### 3.2 Efficiency of Immobilization and Activity of SiO_2_‐*Rr*ADH Supraparticles

For *Rr*ADH immobilization, silica microparticles (denoted hereafter as SiO_2_ supraparticles) are selected as support material. Their synthesis was presented in earlier publications [42, 54] and is depicted in Figure S6. They display a meso‐ to microporous structure, an almost spherical shape, and sizes of approximately 4 c 2 µm.

**FIGURE 3:**
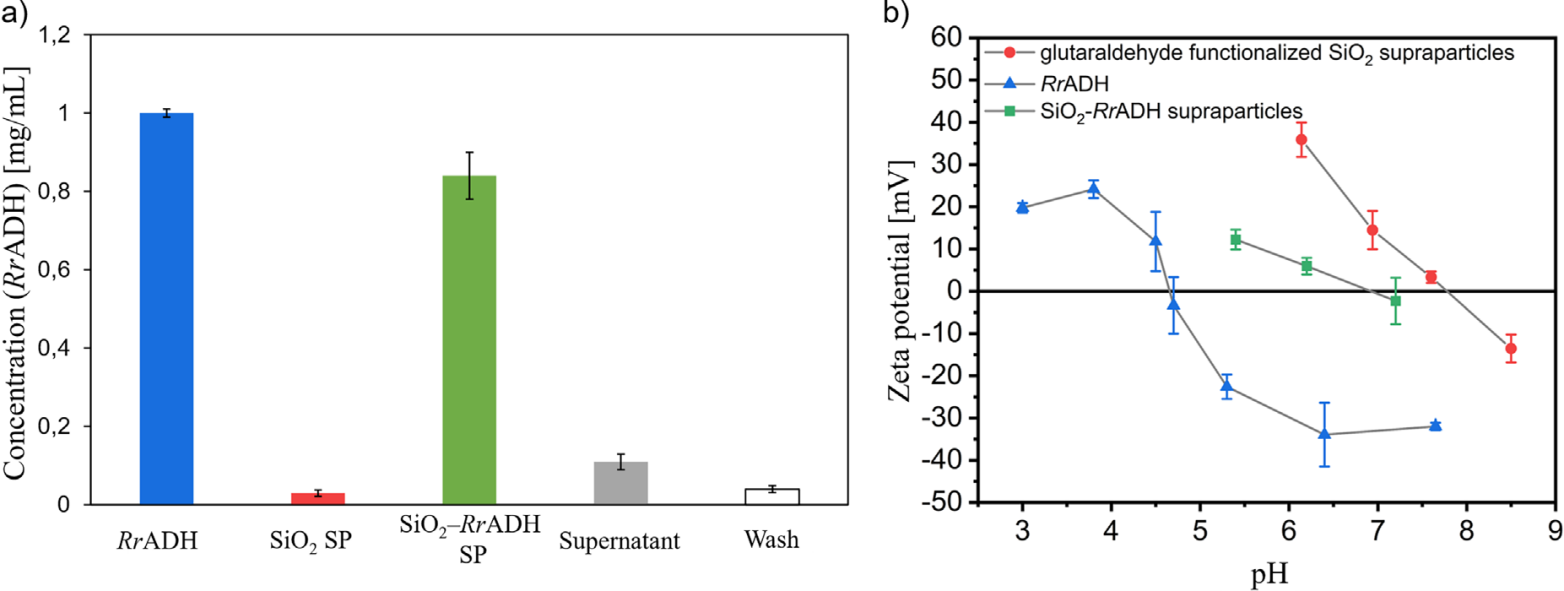
Evaluation of *Rr*ADH immobilization efficiency onto glutaraldehyde‐functionalized SiO_2_ supraparticles. (a) Spectrophotometric quantification at 280 nm, showing concentrations of protein in the load (*Rr*ADH), non‐immobilized control particles (SiO_2_ SP), immobilized particles (SiO_2_‐*Rr*ADH SP), supernatant and wash fractions. (b) Zeta potential measurements of glutaraldehyde‐functionalized SiO_2_ supraparticles, *Rr*ADH, and SiO_2_‐*Rr*ADH supraparticles as a function of pH. The error bars represent the standard deviation of three independent experimental datasets (*n* = 3). *Rr*ADH, *Rhodococcus ruber*.

Spectrophotometric analysis revealed that approximately 85% of the loaded *Rr*ADH was successfully immobilized onto the glutaraldehyde‐functionalized SiO_2_ supraparticles, with only minor protein loss detected in the supernatant and wash fractions (Figure 3a). Zeta potential measurements reveal a shift of the isoelectric point from roughly 7.8 toward a more acidic pH value of 6.9 indicates the successful immobilization of *Rr*ADH onto the glutaraldehyde‐functionalized SiO_2_ supraparticles, as *Rr*ADH displays its isoelectric point at 4.6 (Figure 3b). Inductively coupled plasma optical emission spectroscopy (ICP‐OES) of *Rr*ADH and SiO_2_‐*Rr*ADH supraparticles showed a *Rr*ADH to SiO_2_ supraparticles weight ratio of 1 to 76 (Table S5). The results are in good agreement with the amount of supraparticles and enzymes used for the immobilization (*Rr*ADH to SiO_2_ supraparticles ratio of 1 to 100).

**FIGURE 4:**
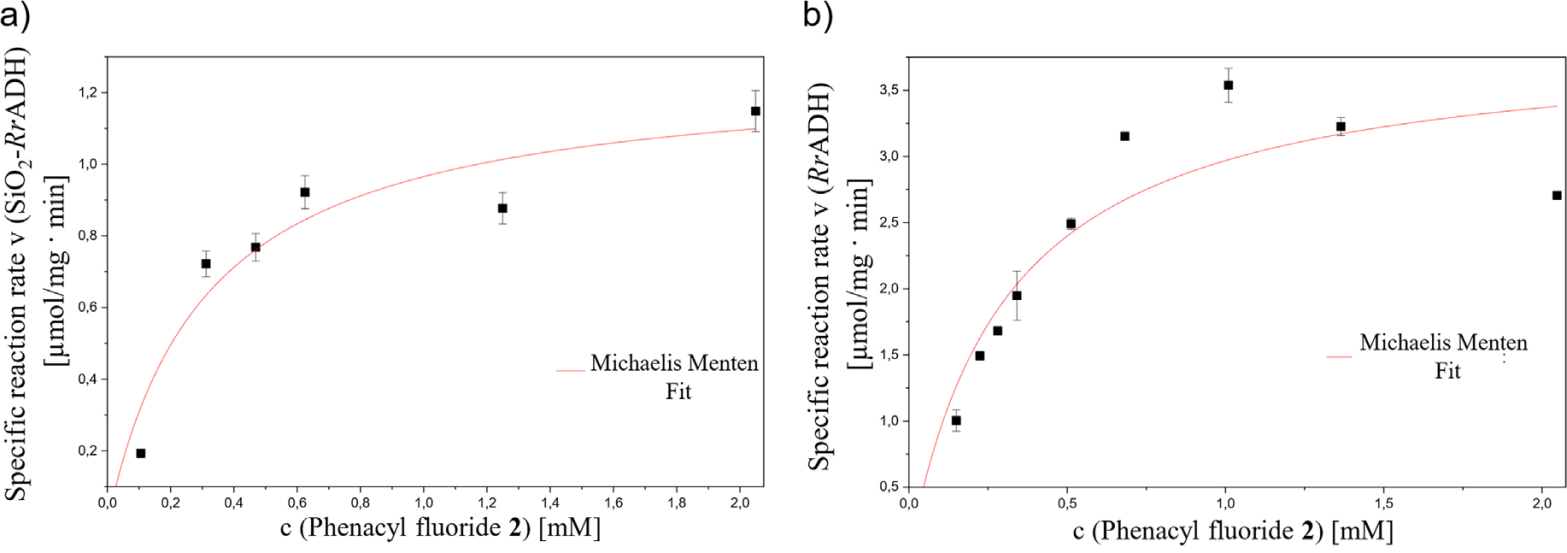
Reaction rates for the reduction of phenacyl fluoride **2** in dependence of the starting concentration of phenacyl fluoride **2** (up to 2 mM), using SiO_2_‐*Rr*ADH supraparticles (a) and *Rr*ADH (b). In order to determine the initial reaction rates, only the initial linear portions of the reaction progress curves were considered (for further details, see Supporting Information). The error bars represent the standard deviation of the mean of two independent experimental datasets (*n* = 2). *Rr*ADH, *Rhodococcus ruber*.

Reaction rates, using SiO_2_‐*Rr*ADH supraparticles, are determined analogously to previously described kinetic studies utilizing free *Rr*ADH within a low substrate concentration window up to 2 mM and are subsequently compared (Figure 4). For this comparison, data points of the kinetic studies, using *Rr*ADH (Figure 2d), are used. Owing to the heterogeneous system, gas chromatography is used for the determination of phenacyl fluoride **2** depletion, instead of UV/VIS spectroscopy (for further details, see SI). For comparison with the free enzyme, an enzyme concentration of 5 µg/mL immobilized *Rr*ADH is used, based on a theoretical amount of loaded enzyme of 1:100 weight ratio (enzyme:supraparticles). This yielded a ν_max_ of 1.27 ± 0.14 µmol/mg•min and a *K_m_* value of 0.31 µmol/mg•min ± 0.11, using SiO_2_‐*Rr*ADH supraparticles (Figure 4a).

The overall observed decrease in reaction rate compared to the homogeneous reaction employing free *Rr*ADH (Figures 4b), despite a suggested, comparable amount of catalytic material, is attributed to the nonquantitative immobilization of *Rr*ADH during the preparation of the SiO_2_‐*Rr*ADH supraparticles. Furthermore, the nonspecific immobilization strategy, involving covalent bonding between random lysine residues of *Rr*ADH and glutaraldehyde functional groups on the silica surface, may compromise enzyme accessibility. This random orientation could lead to partial or complete inaccessibility of the active site, thereby reducing the effective number of catalytically active enzyme molecules. Nevertheless, the immobilization approach is deemed sufficiently effective, as evidenced by the relatively high reaction rates achieved in the enantioselective reduction of phenacyl fluoride **2**.

### 3.3 Activity of *Rr*ADH and SiO_2_‐*Rr*ADH Supraparticles With Recycling of NADH

Following the successful immobilization of *Rr*ADH onto silica supraparticles, the catalytic activity of the resulting SiO_2_‐*Rr*ADH supraparticles is investigated in a NAD^+^/isopropanol‐based system for in situ NADH regeneration. In this setup, a catalytic amount of NAD^+^ (0.1 mM) is employed as a cost‐effective alternative to the stoichiometric addition of NADH. Regeneration of NADH is achieved through the addition of isopropanol (400 mM) in excess, which is oxidized to acetone by *Rr*ADH. Addition of isopropanol not only facilitates efficient cofactor recycling but also serves to shift the reaction equilibrium towards the formation of (*R*)‐2‐fluoro‐1‐phenylethanol **3**, while improving the solubility of both substrate and product. As only a minimal amount of the costly cofactor is required, this system offers considerable potential for cost‐efficient biocatalytic reductions.

A series of experiments is conducted in the initial phase of the study, employing both SiO_2_‐*Rr*ADH nanoparticles and supraparticles with a comparable amount of active enzyme (20 µg/mL, for further details, see Table S7). The determination of yields and ee values is achieved through the utilization of chiral GC (for further details, see SI). The reaction is found to run smoothly under the standard conditions selected for the reaction, thus enabling the reduction of phenacyl fluoride **2** to (*R*)‐2‐fluoro‐1‐phenyl ethanol **3** with excellent ee (> 99.9%) throughout all experiments, while the usage of SiO_2_‐*Rr*ADH supraparticles enables a slightly faster conversion of phenacyl fluoride **2** (Figure S8).

**FIGURE 5:**
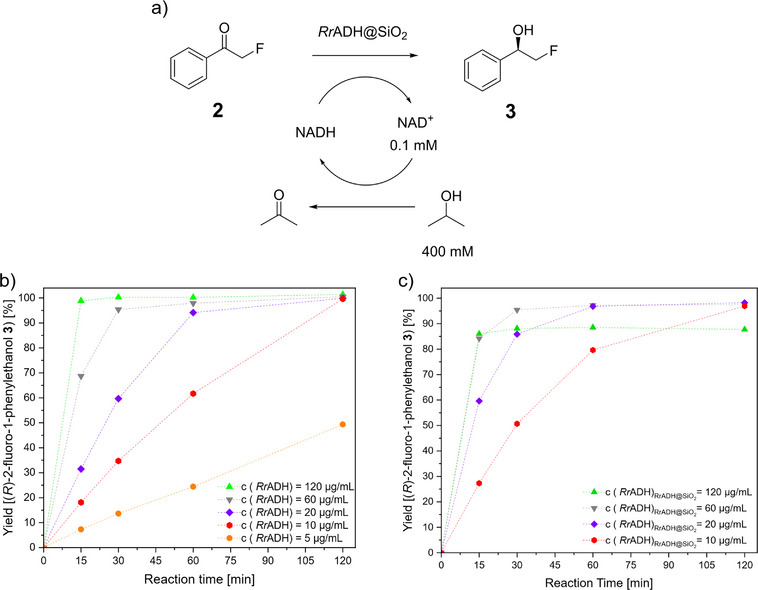
(a) Enantioselective reduction of phenacyl fluoride **2** to (*R*)‐2‐fluoro‐1‐phenylethanol **3**, using *Rr*ADH or SiO_2_‐*Rr*ADH supraparticles as catalyst. NAD^+^ (*c* = 0.1 mM) is used, and the reducing agent NADH is generated in situ by oxidation of isopropanol (400 mM) to acetone. (b) Time‐dependent course of (*R*)‐2‐fluoro‐1‐phenylethanol **3** yield using different concentrations of *Rr*ADH from 120 to 5 µg/mL. (c) Time‐dependent course of (*R*)‐2‐fluoro‐1‐phenylethanol **3** yield using different amounts of SiO_2_‐*Rr*ADH supraparticles from 120 to 10 µg/mL of immobilized *Rr*ADH. *Rr*ADH, *Rhodococcus ruber*.

Activity of *Rr*ADH and SiO_2_‐*Rr*ADH supraparticles in dependency on the amount of utilized catalyst is analyzed in the NAD^+^/isopropanol system (Figure 5).

Using 10 µg/mL of free *Rr*ADH, complete conversion of a 30 mM phenacyl fluoride **2** solution is achieved within 2 h of reaction time (Figure 5b). As expected, increasing the enzyme concentration up to 120 µg/mL significantly reduces the reaction time, resulting in quantitative conversion within 15 min of reaction time. Comparable results are obtained using SiO_2_‐*Rr*ADH supraparticles (Figure 5c). At an immobilized enzyme concentration equivalent to 10 µg/mL *Rr*ADH, a 95% yield of (*R*)‐2‐fluoro‐1‐phenylethanol **3** is achieved within 2 h of reaction time, with excellent enantioselectivity (ee > 99.9%). Higher catalyst loadings further reduce the required reaction time for equivalent conversions.

Notably, elevating the reaction temperature to 35 °C in reactions catalyzed by SiO_2_‐*Rr*ADH supraparticles did not lead to a substantial increase in either reaction rate or yield (Figure S8).

Collectively, these results demonstrate the effectiveness of the NAD^+^/isopropanol regeneration system for both free and immobilized *Rr*ADH, providing a robust and scalable approach for enantioselective ketone reduction.

### 3.4 Storage and Operational Stability of SiO_2_‐*Rr*ADH Supraparticles

For the application of heterogeneous biocatalysts for the synthesis of fine chemicals, with a particular emphasis on long‐term scaled‐up productions, storage stability is exceptionally important. To assess this, the performance of SiO_2_‐*Rr*ADH supraparticles is evaluated in an early study, after 12 months of storage at a temperature of −20 °C.

**FIGURE 6:**
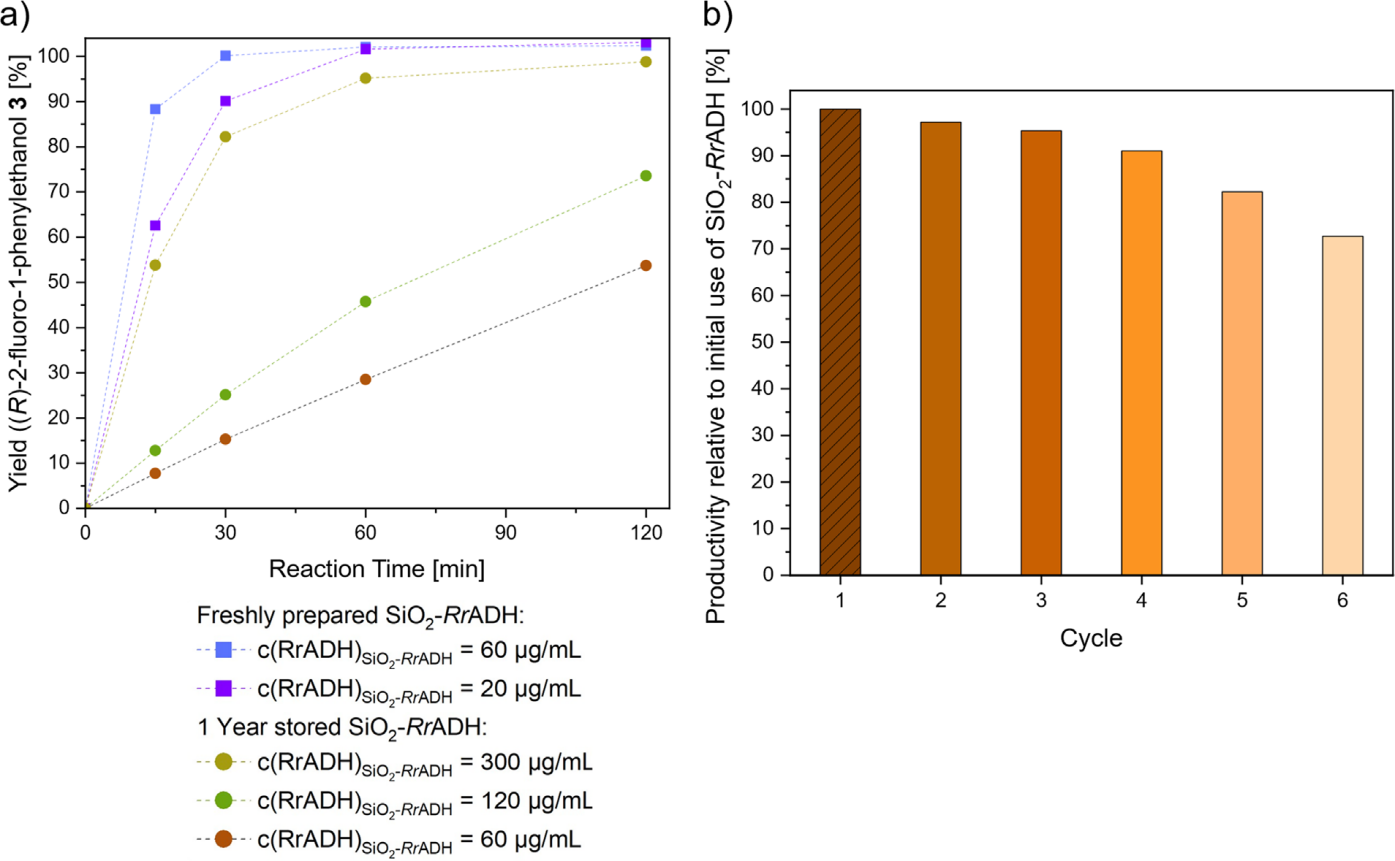
(a) Time‐dependent yield of (*R*)‐2‐fluoro‐1‐phenylethanol **3** obtained with varying concentrations of freshly prepared SiO_2_‐*Rr*ADH supraparticles compared to SiO_2_‐*Rr*ADH supraparticles stored for 1 year at ‐20°C storage temperature. (b) Recyclability of SiO_2–_RrADH supraparticles evaluated over six consecutive reaction cycles for the reduction of phenacyl fluoride **2** to (*R*)‐2‐fluoro‐1‐phenylethanol **3**. Yields are normalized to those obtained with a first‐time use of SiO_2_‐*Rr*ADH supraparticles. For further details, see SI. *Rr*ADH, *Rhodococcus ruber*.

Compared to freshly prepared supraparticles, a significant decline in catalytic activity is observed for the reduction of phenacyl fluoride **2**. Utilizing a catalyst concentration of 60 µg/mL (corresponding to the amount of immobilized *Rr*ADH), a reaction time of 30 min results in a 15% yield for (*R*)‐2‐fluoro‐1‐phenylethanol **3**, whereas a freshly prepared stock under identical conditions resulted in a quantitative conversion (Figure 6a). To match the initial activity of freshly prepared SiO_2_‐*Rr*ADH supraparticles (20 µg/mL of *Rr*ADH), it is necessary to use a 15‐fold higher concentration (300 µg/mL) of the stored catalyst after 1 year. The loss of activity after long‐term storage may be attributed to several factors, including gradual conformational changes, slow hydrolysis of imine (Schiff base) linkages, and physicochemical changes of the carrier material. A detailed investigation of the deactivation mechanisms would require dedicated studies and is beyond the scope of the present work. However, these results indicate that, despite enzyme deactivation upon prolonged storage, the immobilized *Rr*ADH retains a moderate level of catalytic activity, highlighting the potential for storage and reusability, albeit with a trade‐off in efficiency over time.

The recyclability of SiO_2_‐*Rr*ADH supraparticles is evaluated through repeated use in the reduction of phenacyl fluoride **2**, followed by recovery of the catalyst via centrifugation. After each reaction cycle, the particles are subjected to thorough washing with MOPS buffer (for procedural details, see SI) and reused for subsequent reactions. This cycle is repeated six times. Product yields of (*R*)‐2‐fluoro‐1‐phenylethanol **3** are determined for each cycle and normalized to the yield obtained with an initial reaction cycle (Figure 6b for further details, see SI). The results indicate a gradual decline in relative activity with each reuse; however, more than 90% of the initial activity is retained through four cycles, and 75% activity remains after the sixth cycle. It is important to note that complete recovery of the supraparticles cannot be guaranteed due to catalyst loss during the centrifugation and washing steps. Therefore, the observed decrease in catalytic performance may be attributed, at least in part, to loss of catalyst material rather than deactivation of the enzyme. Overall, these findings demonstrate that SiO_2_‐*Rr*ADH supraparticles exhibit promising recyclability properties, supporting their potential for application in sustainable, heterogeneous biocatalysis.

*Rr*ADH exhibits remarkable physicochemical properties, including substantial tolerance toward organic co‐solvents [45], which facilitates the solubilization of hydrophobic substrates and supports the use of isopropanol for in situ NADH regeneration, as demonstrated in previous sections. The stability of enzymes in the presence of organic solvents is generally attributed to a high content of hydrophobic amino acid residues and robust intramolecular interactions, which together stabilize the protein's conformation [53, 55]. Structural investigations of *Rr*ADH, conducted by Gruber et al. [45], suggest that its solvent tolerance is primarily associated with salt bridges between the protein monomers. These intermolecular interactions at the monomer‐monomer interface are proposed to play a key role in maintaining both the tertiary and quaternary structure of the enzyme, thereby preserving catalytic function under challenging reaction conditions.

To establish the envisioned two‐step synthesis of (*R*)‐2‐fluoro‐1‐phenylethanol **3**, it is essential to integrate both the decarboxylative fluorination of 3‐oxo‐3‐phenylpropanoic acid **1** and the subsequent enantioselective reduction into a combined process. As previously described [43], the fluorination step is carried out in a water‐methanol mixture, necessitating that the subsequent reduction step using SiO_2_‐*Rr*ADH supraparticles is also compatible with the presence of methanol as co‐solvent. However, a marked decline in enzymatic activity is observed during the reduction of phenacyl fluoride **2** at methanol concentrations ≥10% v/v (Figure S9). In particular, the use of 30% v/v methanol leads to a pronounced reduction in catalytic efficiency, yielding only 35% of (*R*)‐2‐fluoro‐1‐phenylethanol **3**. These findings indicate a clear sensitivity of the immobilized enzyme system to elevated concentrations of methanol. As a result, methanol concentrations exceeding 15% v/v by far are deemed unsuitable for this tandem process, both from a practical and process‐optimization standpoint, thereby necessitating careful adjustment of solvent composition to balance reaction compatibility and enzymatic performance.

To further explore the applicability of the system, the tolerance of SiO_2_‐*Rr*ADH supraparticles toward organic solvents is assessed by conducting the reduction of phenacyl fluoride **2** in a three‐phase (liquid–liquid–solid) system. In this setup, the substrate phenacyl fluoride **2** is dissolved in the organic phase (cyclohexane or ethyl acetate), and the SiO_2_‐*Rr*ADH supraparticles are suspended in an aqueous MOPS buffer phase (Figure S10a). The reaction proceeds efficiently using cyclohexane, demonstrating that SiO_2_‐*Rr*ADH supraparticles remain catalytically active in the presence of cyclohexane (Figure S10b). However, due to the low distribution coefficient of phenacyl fluoride **2** in the cyclohexane‐water system, a significant portion of the substrate remains in the organic phase. This limits substrate accessibility to the hydrophilic, water‐suspended enzyme particles and consequently results in a reduced reaction rate. This effect is even more pronounced when ethyl acetate is used as the organic solvent (Figure S10c), leading to further decreases in catalytic activity under otherwise identical conditions.

In summary, while the enzyme displays tolerance toward hydrophobic solvents, the overall reaction rate in the three‐phase system is significantly lower than that observed in a biphasic aqueous suspension, primarily due to substrate distribution effects that limit catalyst‐substrate interaction.

### 3.5 Enantioselective Reduction of Phenacyl Fluoride **2** in Continuous Flow Mode With *Rr*ADH and SiO_2_‐*Rr*ADH Supraparticles

Following the comprehensive evaluation of *Rr*ADH and SiO_2_‐*Rr*ADH supraparticles in the enantioselective batch reduction of phenacyl fluoride **2**, the process is transferred to a continuous flow setup as a preparatory step towards establishing an integrated two‐step continuous flow synthesis, incorporating the decarboxylative fluorination of 3‐oxo‐3‐phenylpropanoic acid **1** as the initial step.

**FIGURE 7:**
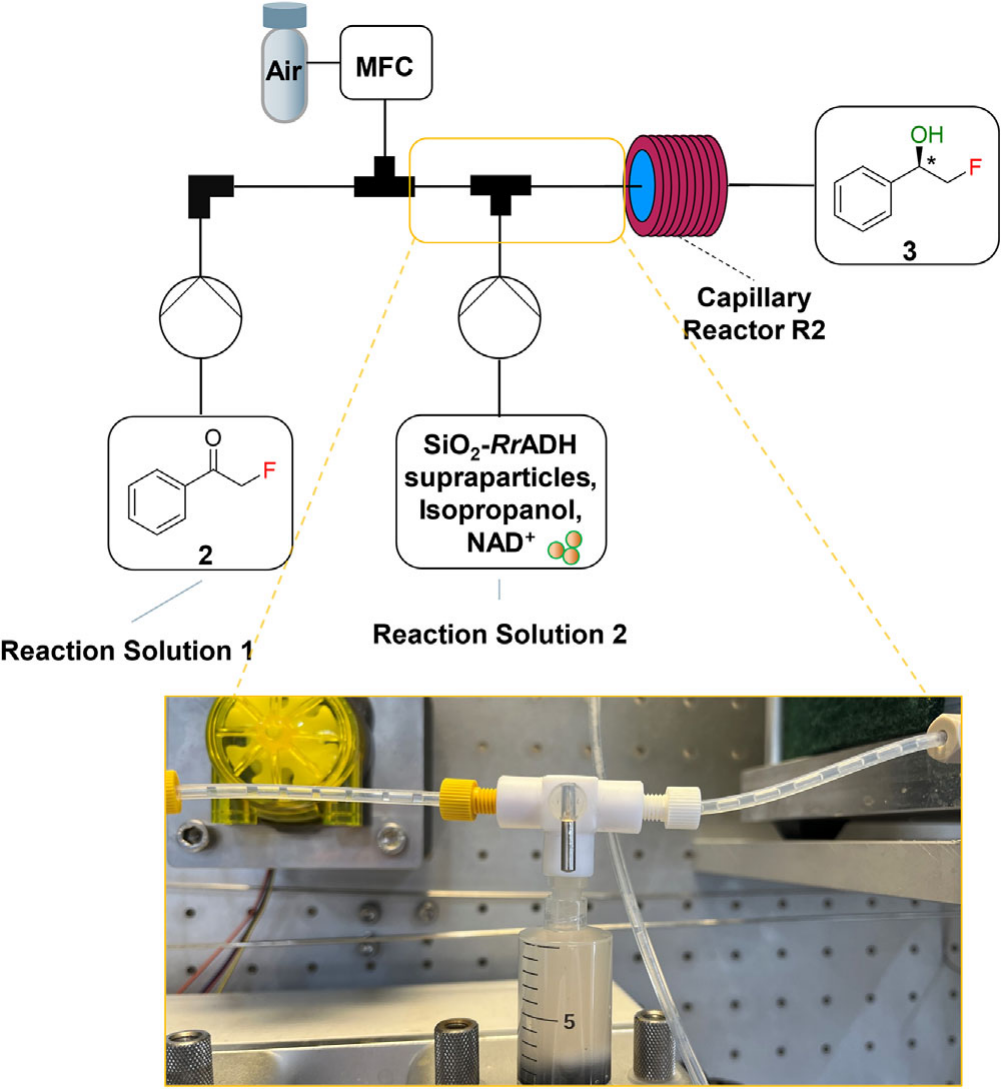
Flow scheme for the enantioselective reduction of phenacyl fluoride **2** with SiO_2_‐*Rr*ADH supraparticles. A well‐defined slug flow is created by introducing air to ensure a continuous transportation of the supraparticles.

A fluorination reaction mixture (reaction solution 1) containing phenacyl fluoride **2** is first prepared in batch and then introduced into the continuous flow system (Figure 7). A second solution (reaction solution 2) comprises either free *Rr*ADH or SiO_2_‐*Rr*ADH supraparticles, along with NAD^+^, isopropanol, and MOPS buffer. The flow setup consists of a syringe pump that continuously doses reaction solution 1, while a mass flow controller provides a precisely regulated flow of synthetic air, resulting in the formation of a gas/suspension slug flow regime. The reduction step is initiated by a second syringe pump, which introduces reaction solution 2 into the system. This pump is equipped with an external magnetic stirring unit, ensuring continuous agitation of the supraparticle suspension within the syringe to prevent sedimentation. The combined gas‐liquid slugs are transported into the capillary reactor R2, consisting of a 1/8‐inch FEP (fluorinated ethylene propylene) capillary with a total volume of 30 mL. The reactor temperature is maintained at 25 °C using an external thermostat. Residence time is precisely controlled by adjusting the flow rates of the individual flows.

The use of methanol as a cosolvent appears to improve slug flow dynamics, likely due to a reduction in contact angle, resulting in stable, well‐formed slugs where air segments are enveloped by a thin aqueous film (Figure S11). This stable flow regime reduces the risk of clogging and provides the foundation for a robust, long‐term, continuous operation. Efficient segment distribution and toroidal mixing currents within the slugs ensured uniform dispersion of the supraparticles, facilitating an efficient continuous flow biocatalytic process.

For the continuous flow experiments, the flow rates of both reaction solutions as well as the synthetic air flow are adjusted to achieve residence times equivalent to those used in batch experiments with a reaction time of 1 h. A catalyst concentration of 60 µg/mL (based on the theoretical amount of *Rr*ADH) is used throughout the continuous flow experiments.

The application of both free *Rr*ADH and SiO_2_‐*Rr*ADH supraparticles within the flow system reproduced the high performance observed under batch conditions (Table 1), achieving near‐quantitative conversions of phenacyl fluoride **2** to (*R*)‐2‐fluoro‐1‐phenylethanol **3**. Importantly, the enantioselectivity remained consistently high, with *ee* values exceeding 99.9% for both catalyst systems. These findings confirm the robustness and compatibility of the supraparticles with a capillary reactor, providing a valuable and modular alternative to batch processing while preserving catalytic performance.

**TABLE 1:** Reaction details and results for the enantioselective reduction of phenacyl fluoride **2** in batch and continuous flow mode, using free *Rr*ADH and SiO_2_‐*Rr*ADH supraparticles.

Entry	Mode	Catalyst	c_0_, _Phenacyl fluoride 2_ (mM)	*c _Rr_*_ADH_ (µg/mL)	Reaction time or Residence time [min]	Yield (%)
1	Batch	*Rr*ADH	30	60	60	100
2	Flow^a^	*Rr*ADH	30	60	60	99
3	Batch	SiO_2_‐ *Rr*ADH	30	60	60	95
4	Flow^a^	SiO_2_‐ *Rr*ADH	30	60	60	98

^a^ Parameters used for continuous flow reactions: Flow rate (air) = 0.3 mL/min; Flow rate (Reaction solution 1) = 0.1 mL/min; Flow rate (Reaction solution 2) = 0.1 mL/min; V_Reactor_ = 30 mL; T = 25°C.

### 3.6 Two‐Step Continuous Flow Synthesis of (*R*)‐2‐Fluoro‐1‐Phenyl Ethanol With SiO_2_‐*Rr*ADH Supraparticles

The decarboxylative monofluorination of β‐keto acid **1** and the subsequent enantioselective reduction of the intermediate phenacyl fluoride **2** using SiO_2_‐*Rr*ADH supraparticles are successfully transferred into a combined continuous flow process. The setup for the fluorination step is adapted from a previously published work [43].

**FIGURE 8:**
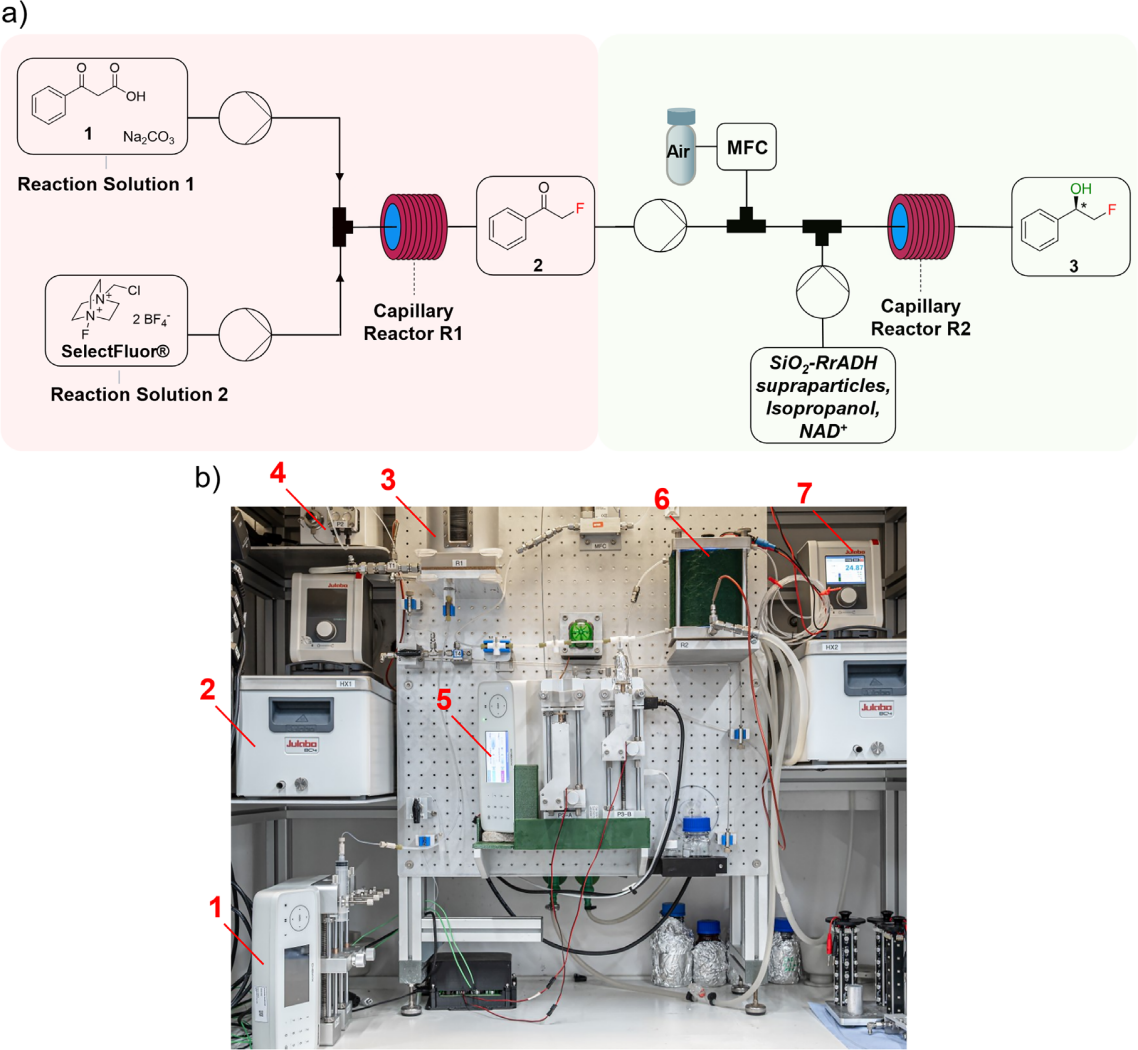
Flow scheme for the consecutive two‐step process, including the decarboxylative fluorination of 3‐oxo‐3‐phenylpropanoic acid **1** to the intermediate phenacyl fluoride **2** and the subsequent enantioselective reduction with SiO_2_‐*Rr*ADH supraparticles, yielding (*R*)‐2‐fluoro‐1‐phenyl ethanol **3**. **1**: Syringe pump 1 and 2, **2**: Thermostat 1, **3**: Capillary reactor R1, **4**: HPLC pump with reservoir, **5**: Syringe pump 3, **6**: Capillary reactor R2, **7**: Thermostat 2.

The continuous flow platform (Figures 8 and S12) consists of two capillary reactors, R1 (Figure S13) and R2, where each reactor is constructed from coiled 1/8‐inch FEP tubing, wound around cylindrical cores and temperature‐controlled by two separate thermostats. Two distinct reaction solutions, one containing the β‐keto acid **1** and sodium carbonate, the other SelectFluor as fluorination reagent, are delivered by syringe pumps and combined via an interconnected Y‐piece, initiating the fluorination reaction. The reaction mixture is then introduced into reactor R1, operated at 60 °C, with a residence time of 60 min.

After exiting R1, the produced intermediate solution of phenacyl fluoride **2** is collected in a reservoir and continuously transferred to the second section of the lab plant using an HPLC pump. At this stage, a slug flow regime is established by introducing a synthetic air flow. In parallel, a suspension containing SiO_2_‐*Rr*ADH supraparticles, NAD^+^, and isopropanol in MOPS buffer is introduced into the system via a second syringe pump. Upon mixing both the reaction solution of phenacyl fluoride **2** and the catalyst suspension in a 1:1 volume ratio, a final concentration of 15 mM phenacyl fluoride **2** and a methanol concentration of approximately 16.7% v/v is obtained and the enzymatic reduction step is initiated. The combined flow is directed into capillary reactor R2, which is maintained at 25°C, providing a residence time of 60 min. The resulting suspension is continuously collected, and the reaction is quenched immediately upon sampling by the addition of ethyl acetate in excess, followed by drying over magnesium sulfate. Yields and enantiomeric excesses are determined by chiral gas chromatography, confirming the successful implementation of the integrated two‐step continuous flow process.

The production of (*R*)‐2‐fluoro‐1‐phenylethanol **3** in the continuous flow system requires a defined time span to reach steady‐state conditions. The total product yield increases progressively over the course of the reaction until a 90% yield is reached after approximately 70 min of operation (Figure S14), which corresponds to about 2.5 reactor residence times.

The determined yield is in excellent agreement with the expected outcome based on the performance of the individual reaction steps. Specifically, a 91% yield is obtained for the decarboxylative fluorination of 3‐oxo‐3‐phenylpropanoic acid **1** [43], while the subsequent enantioselective reduction of phenacyl fluoride **2** proceeds with quantitative conversion under the applied conditions.

## 4 Conclusion

In this study, *Rr*ADH purified in a plant‐based cell‐free system was demonstrated to exhibit sufficient catalytic performance in the enantioselective reduction of phenacyl fluoride to *R*‐2‐fluoro‐1‐phenylethanol, affording quantitative yields and exceptional enantiomeric excess (ee > 99.9%). Comprehensive kinetic investigations were conducted, and the enzyme was successfully immobilized on glutaraldehyde‐functionalized silica supraparticles, achieving a high immobilization efficiency. Comparative analysis between the free and immobilized enzyme revealed that the SiO_2_‐*Rr*ADH supraparticles retained remarkable catalytic activity while enabling efficient in situ cofactor regeneration in a NAD^+^/isopropanol system. Preliminary stability studies further indicated promising long‐term storage stability and recyclability of the heterogeneous biocatalyst. Finally, the SiO_2–_RrADH supraparticles were successfully implemented in a continuous flow setup, enabling the continuous synthesis of *R*‐2‐fluoro‐1‐phenylethanol via a two‐step process comprising the decarboxylative fluorination of 3‐oxo‐3‐phenylpropanoic acid followed by the enantioselective reduction of the intermediate phenacyl fluoride. In conclusion, the originality of this work lies not in the individual methodological components but in their deliberate integration into a continuous two‐step flow system and the experimental validation of its performance under practical operating conditions. Future work will focus on the continuous operation and recyclability of the SiO_2_‐*Rr*ADH supraparticles, as well as the extension of this approach to structurally related substrates, paving the way for the sustainable synthesis of chiral fluorinated building blocks via assisted biocatalysis, including a simplified downstream process with immobilized enzymes in a continuous flow process.

## Conflicts of Interest

The authors declare no conflicts of interest.

## Supporting information




**Supporting File 1**: chem70748‐sup‐0001‐SuppMat.pdf.
