# Supplementary material for: Two‐Step Synthesis of a Chiral Fluorinated Alcohol With Silica‐Supported Enzyme RrADH in Batch and Continuous Flow Mode
Source: Chemistry. 2026 Feb 9;32(16):e03304. doi: 10.1002/chem.202503304 (PMC13109679; doi:10.1002/chem.202503304)
Supplement: Supplementary file 1 — Supporting File 1: chem70748‐sup‐0001‐SuppMat.pdf. [file CHEM-32-e03304-s001.pdf]

Supporting Information

Two-Step Synthesis of a Chiral Fluorinated Alcohol with Silica-Supported Enzyme *Rr*ADH in Batch and Continuous Flow Mode

Egzon Cermjani[a, b,†], Greta Nölke[c,†], Stephano Di Fiore[c], Christoph Deckers[a], Bettina Herbig[d], Doris Hanselmann[d], Susanne Wintzheimer[d], Thomas H. Rehm[a]**

[a] *Fraunhofer Institute for Microengineering and Microsystems IMM, Carl-Zeiss-Strasse 18-20, 55129 Mainz, Germany.*

[b] *Johannes Gutenberg-University Mainz, Department of Chemistry, Duesbergweg 10-14, 55128 Mainz, Germany.*

[c] *Fraunhofer Institute for Molecular Biology and Applied Ecology IME, Forckenbeckstraße 6, 52074 Aachen, Germany*

[d] *Fraunhofer Institute for Silicate Research ISC, Neunerplatz 2, 97082 Würzburg, Germany*

*Corresponding Authors: egzon.cermjani@imm.fraunhofer.de, thomas.rehm@imm.fraunhofer.de

[†] These authors contributed equally to this work.

1. General Information

Materials and Hardware.

All chemicals are purchased from commercial vendors and used as arrived unless otherwise mentioned.

Table S 1. Hardware components installed for continuous flow reactions.

Hardware component	Specifications/Model	Supplier
Syringe pump	Model: Fusion 4000 X	Chemyx
Thermostat	F10 with HC E07 – control unit	Julabo GmbH
Tubing	1/8” FEP-capillary	Bolender GmbH
T-junctions and connectors		IDEX Health & Science LLC
HPLC Pump	Model: P4.1S	KNAUER Wissenschaftliche Geräte GmbH

Thin-layer chromatography.

Qualitative thin-layer chromatography is conducted on SilG/UV254 silica gel plates with a layer thickness of 0.25 mm, procured from Machery Nagel & Co., Düren. The resulting chromatograms are initially analyzed under a UV lamp (254 nm).

Gas chromatography.

Gas chromatography with a flame ionization detector (FID) was performed using the Shimadzu GC 2030. Samples were extracted with ethyl acetate and dried over $MgSO_4$. The organic extract is then diluted with ethyl acetate (1:20) prior to GC measurements. For detailed information about the GC-FID program, see: Table S 2.

Table S 2. Settings for GC measurements.

Machine	Shimadzu GC 2030
Column	Agilent CP-Chirasil-Dex<br>Length: 25 m<br>Inner diameter 0.32 mm<br>Film thickness: 0.25 µm
Detector	FID<br>Setpoint: 230 °C<br>Flow: 24 mL/min<br>H_2: 32 mL/min<br>Air: 200 mL/min
Oven temperature	Initial: 100 °C for 3 min<br>1st gradient: 20 °C/min from 100 °C to 130 °C (hold time: 11 min)<br>2st gradient: 15 °C/min from 130 °C to 215 °C (hold time: 1.3 min)
Injector temperature	200 °C
Carrier gas	N_2
Flow rate	1.15 mL/min (column flow), 15.7 mL/min (total flow), 3.0 mL/min (purge flow)
Split ratio	10
Injection	1 µL
Stop time	22.47 min
Solvent	Ethyl acetate
**Retention times**	
Phenacyl fluoride **2**	7.913 min
(*S*)-2-fluoro-1-phenylethanol **3**	12.786 min
(*R*)-2-fluoro-1-phenylethanol **3**	13.430 min

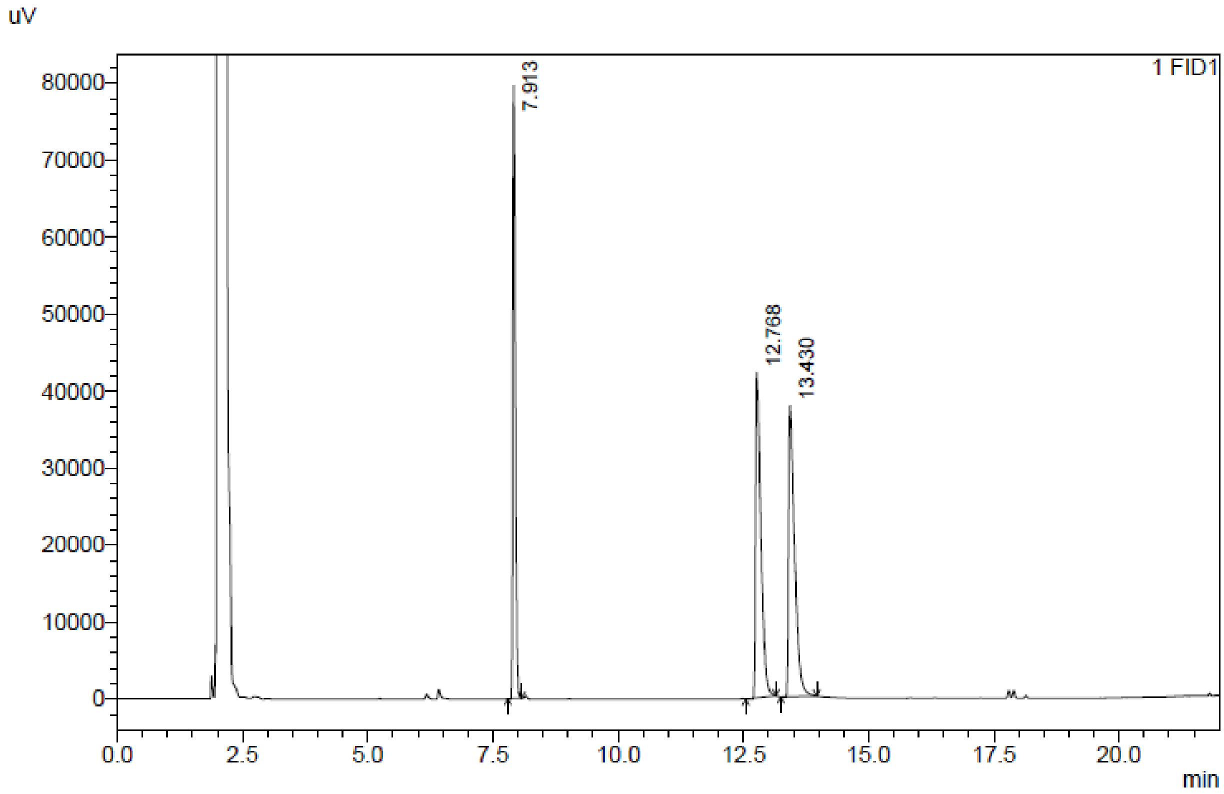

Figure S 1. GC chromatogram of a standard solution in ethyl acetate, containing phenacyl fluoride **2** and both (*R*)- and (*S*)-2-fluoro-1-phenylethanol **3**.

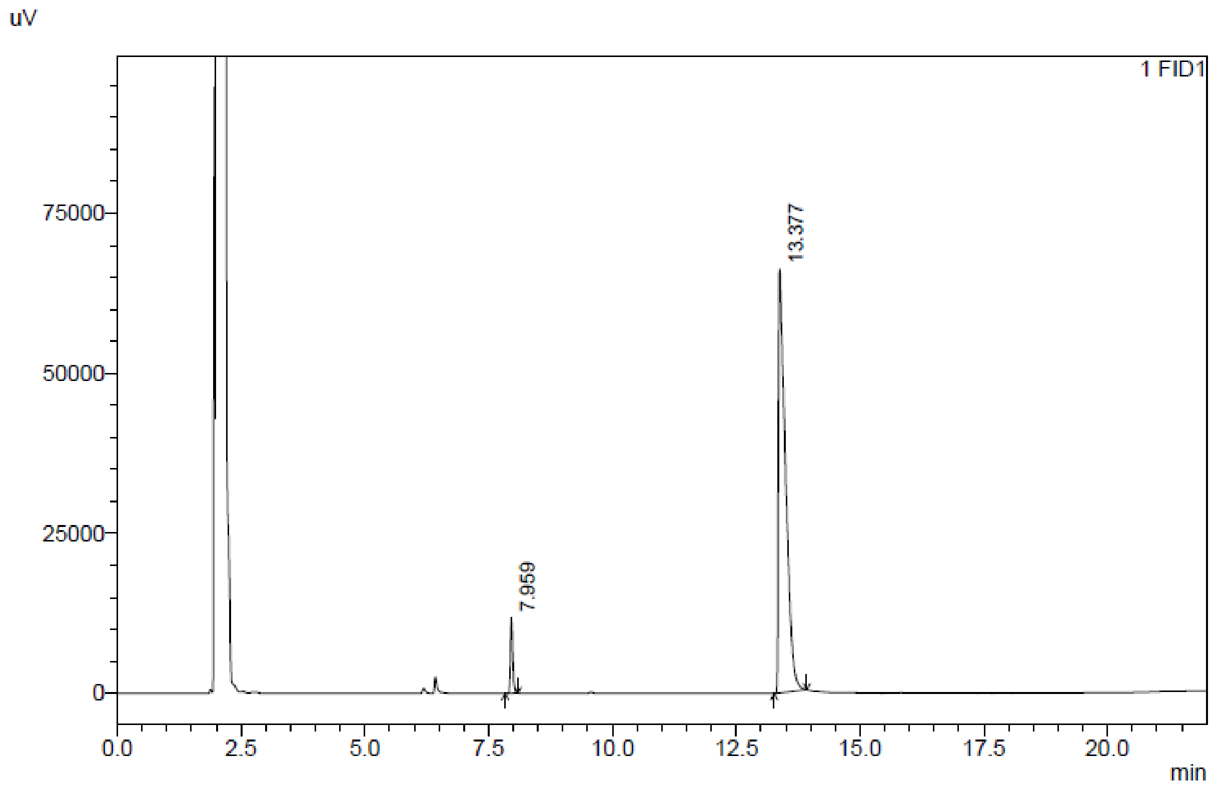

Figure S 2. GC chromatogram of a reaction solution for the enzyme catalyzed reduction of phenacyl fluoride **2**, yielding (*R*)-2-fluoro-1-phenyethanol **3**, extracted by ethyl acetate.

GC-MS.

GC-MS analysis was carried out with a Shimadzu GC-2010 Plus/QP2010 Ultra. Samples were extracted with ethyl acetate. Mesitylene was added as internal standard. For detailed information about the GC program, see: Table S 3.

Table S 3. Settings for GC-MS measurements.

Machine	Shimadzu GC-2010 Plus
Column	Phenomex ZebronTM ZB-5MSi<br>Length: 30 m<br>Inner diameter 0.25 mm<br>Film thickness: 0.25 µm
Detector	Shimadzu QP2010 Ultra<br>Setpoint: 230 °C<br>Interface: 230 °C
Oven temperature	Initial: 70 °C for 2.5 min<br>1st gradient: 10 °C/min from 70 °C to 200 °C (hold time: 0 min)<br>2st gradient: 30 °C/min from 200 °C to 230 °C (hold time: 2.5 min
Injector temperature	230 °C
Carrier gas	Helium
Flow rate	1.28 mL/min (column flow), 13.9 mL/min (total flow), 3.0 mL/min (purge flow), 41.5 cm/sec (linear velocity)
Split ratio	7.5
Stop time	19.0 min
Solvent	Ethyl acetate

**Retention times**	
3-oxo-3-phenyl propanoic acid **1** (detected as acetophenone due to thermal decarboxylation)	6.561 min
Phenacyl fluoride **2**	7.242 min
(*R*)-2-fluoro-1-phenylethanol **3**	7.245 min

NMR spectroscopy.

Nuclear magnetic resonance (NMR) spectra were recorded on an 80 Carbon Ultra NMR spectrometer manufactured by Magritek with Larmor frequencies of 80.22 MHz for ^{1}H-NMR, 75.47 MHz for ^{19}F-NMR and 20.17 MHz for ^{13}C-NMR. High-resolution ^{1}H-, ^{13}C- and ^{19}F-NMR spectra were recorded with an Avance II 400 NMR spectrometer (Bruker Corporation). The spectra were analyzed using the program MestReNova 14.2 by Mestrelab Research. The chemical shifts are given in parts per million (ppm). The solvent residual signal was used as reference signal for ^{1}H and ^{13}C spectra. The fine structures of the signals are indicated with the following abbreviations: ‘s’ for singlet, ‘d’ for doublet, ‘t’ for triplet, ‘m’ for multiplet.

Procedure for the NADH depletion study using *Rr*ADH and UV/VIS spectroscopy

For UV/VIS spectroscopy measurements, a quartz glass cuvette (d = 2 mm) was used. For determination of NADH depletion, a 0.6 mL reaction mixture, containing 0.3 mL NADH solution (c (stock) = 4 mM) in MOPS buffer (50 mM, pH 6.8) and 5 mg/mL of *Rr*ADH (c_{stock} (*Rr*ADH) = 0.187 mg/mL) are mixed together. A phenacyl fluoride solution (c = 22.4 mM) was prepared in MOPS buffer (50 mM, pH 6.8), containing 2 v/v% DMSO and was added to the reaction solution, together with an appropriate amount of MOPS buffer for reaching 0.6 mL of total volume. The cuvette was subsequently put into the UV/VIS spectrometer and spectra ranging 450 nm to 280 nm wavelength were recorded in dependency of the reaction time.

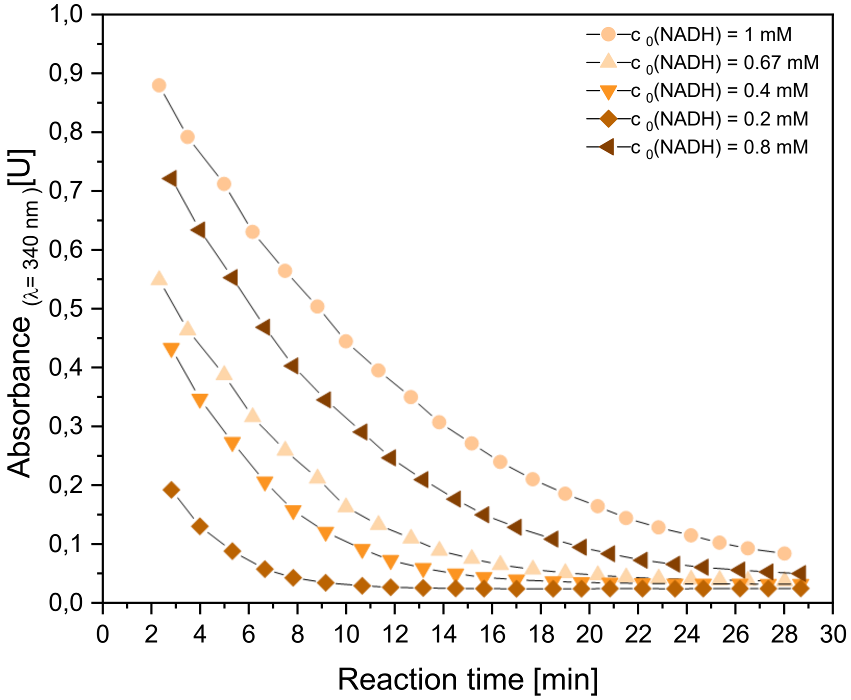

Figure S 3. Depletion of the absorbance of NADH at λ = 340 nm over the course of the reduction of phenacyl fluoride **2**, catalyzed by *Rr*ADH in MOPS buffer (50 mM, pH 6.8, 2% DMSO).

Different starting concentrations of NADH were used. c (*Rr*ADH) = 5 µg/mL. c (phenacyl fluoride **2**) = 4 mM.

Table S 4. Reaction details for the NADH depletion study with *Rr*ADH. T = 25 °C.

#	V_{total}	V_{NADH}	$V_{phenacyl\ fluoride}$	$c_{phenacyl\ fluoride}$	$V_{MOPS\ buffer}$	V_{RrADH} (c_{stock} = 0.187 mg/mL)
1	0.6 mL	0.15 mL (a 8 mM stock was used)	0.4 mL	15.011	0.45 mL	16 µL
2	0.6 mL	0.3 mL	54.8 µL	2.048	0.245 mL	16 µL
3	0.6 mL	0.3 mL	36.5 µL	1.365	0.264 mL	16 µL
4	0.6 mL	0.3 mL	27.0 µL	1.010	0.273 mL	16 µL
5	0.6 mL	0.3 mL	18.2 µL	0.683	0.282 mL	16 µL
6	0.6 mL	0.3 mL	13.7 µL	0.512	0.286 mL	16 µL
7	0.6 mL	0.3 mL	9.1 µL	0.341	0.290 mL	16 µL
8	0.6 mL	0.3 mL	7.5 µL	0.280	0.293 mL	16 µL

9	0.6 mL	0.3 mL	6.0 µL	0.224	0.294 mL	16 µL
10	0.6 mL	0.3 mL	4.5 µL	0.150	0.296 mL	16 µL

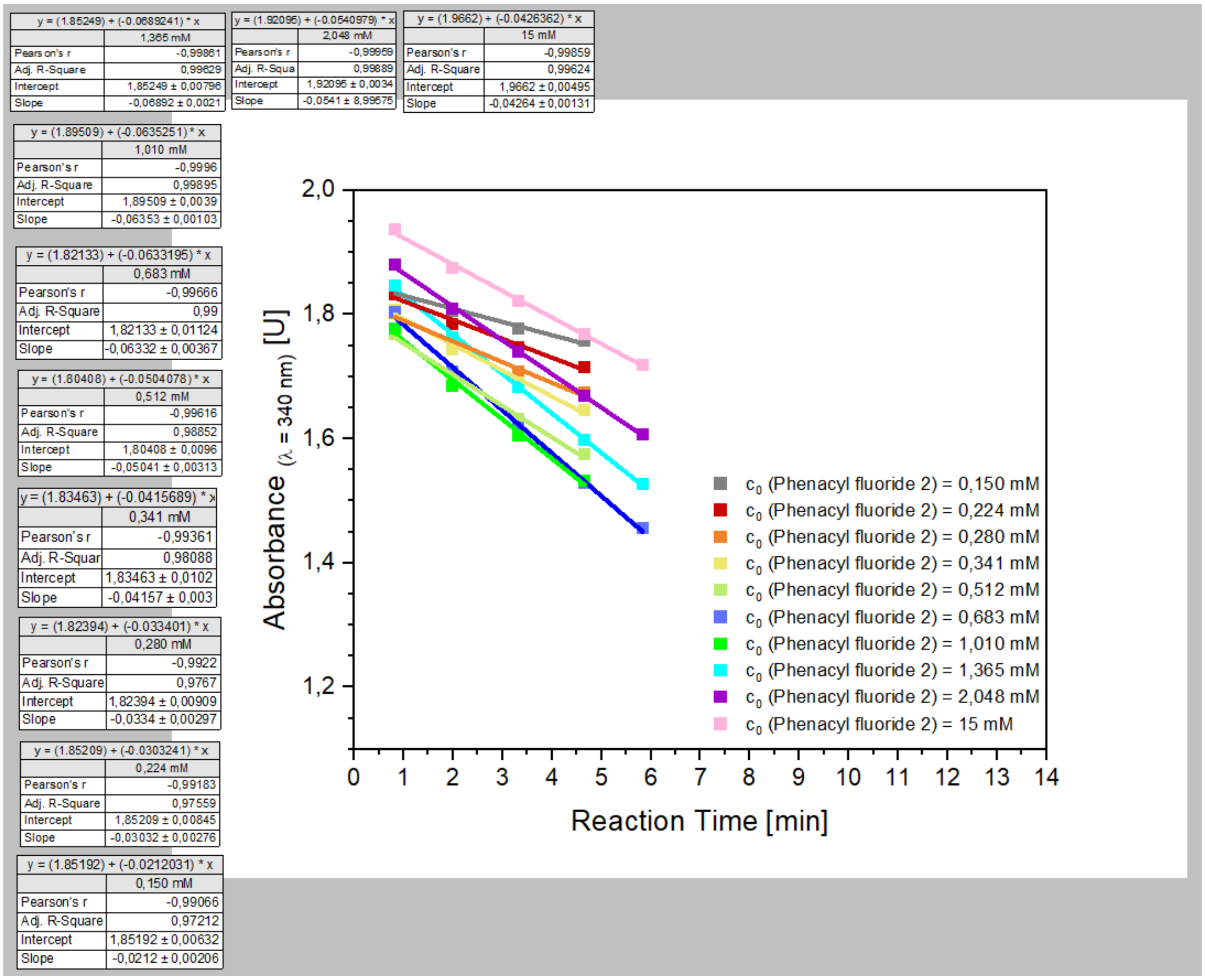

Figure S 4. Linear regression analyses were performed on the experimental data obtained from the biocatalytic reduction of phenacyl fluoride **2** with NADH and *Rr*ADH. For each experimental set, different initial concentrations of phenacyl fluoride **2** were applied. In order to determine the initial reaction rates, only the linear portions of the reaction progress curves were considered.

Specifically, a subset of the early time points was selected for linear fitting to ensure a coefficient of determination (R^2) greater than 0.97. Initial reaction rates are then plotted against the concentration of phenacyl fluoride **2** and nonlinear regression according to the Michaelis–Menten model was performed using the built-in fitting function (Figure S 5) in OriginPro 2025 (Version 10.25).

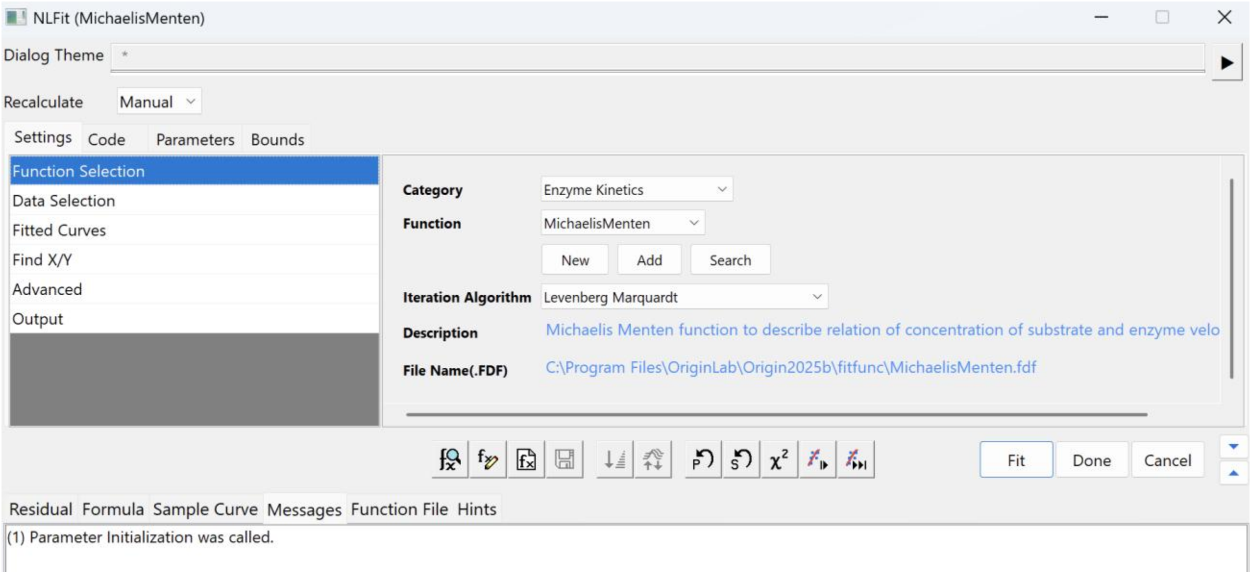

Figure S 5. NLFit (MichaelisMenten) Fitting function in OriginPro 2025 (Version 10.25).

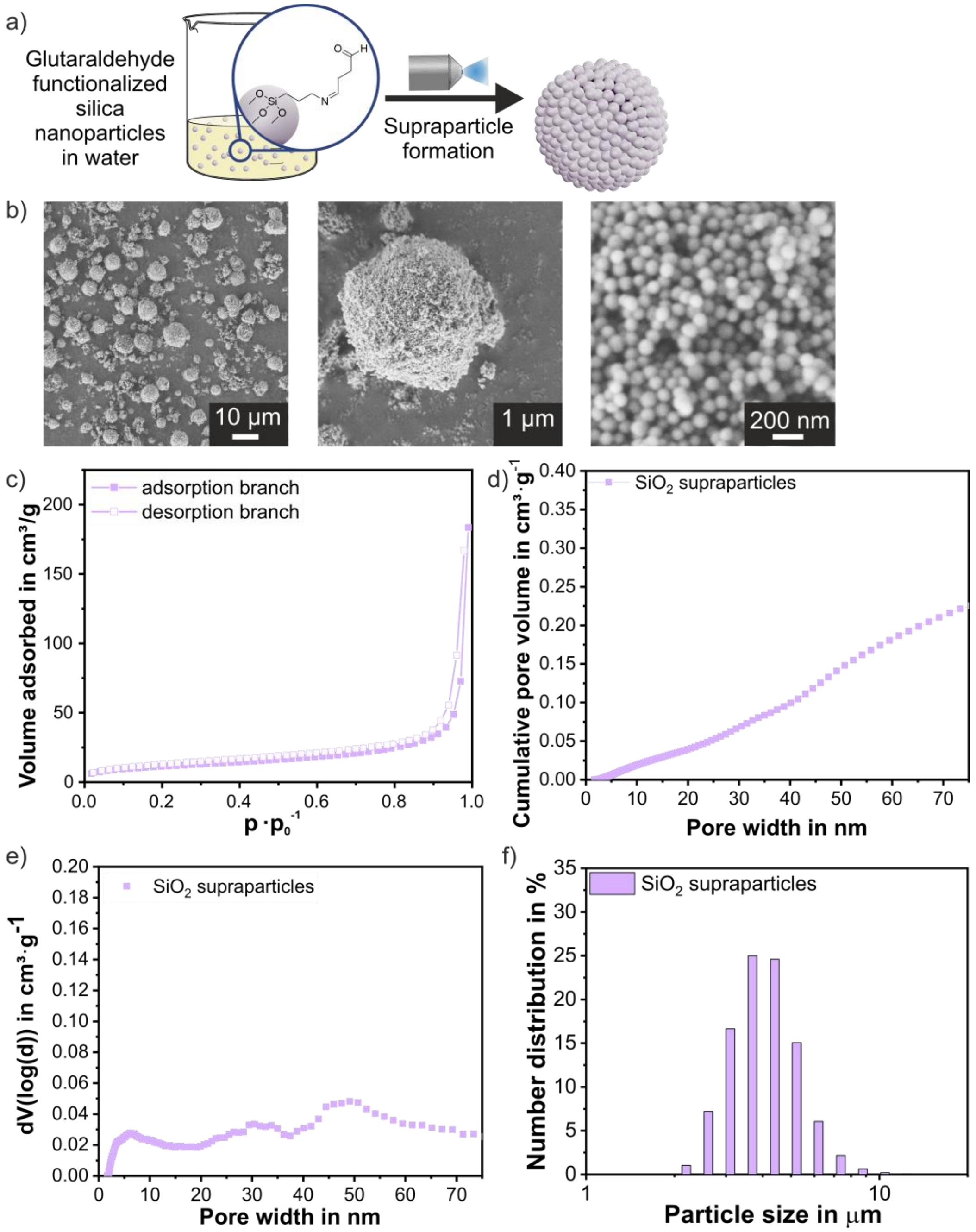

Figure S 6. Schematic representation of SiO_2 supraparticles fabrication using the spray-drying of glutaraldehyde functionalized SiO_2 nanoparticles dispersed in water. b) SEM micrographs showing three different magnifications, and c) N_2 adsorption (full symbols) / desorption (open symbols) isotherms of SiO_2 supraparticles. d) The cumulative pore volume was obtained from NLDFT, and e) the pore size distribution plot was calculated by applying a dedicated NLDFT

method on the adsorption branch of the isotherms, assuming a cylindrical pore shape. f) The number-weighted size distribution of SiO_2 supraparticles measured via laser diffraction. a) to f) reproduced from Herbig et al.[46] under terms of the CC-BY license.

Table S 5. ICP measurements for *Rr*ADH- and SiO_2-*Rr*ADH supraparticles.

**sample**	**Used concentration of sample in mg/L**	**Measured concentration Zn in mg/L**	**Calculated amount Zn per sample in weight-%**	**Calculated amount of *Rr*ADH concentration in mg/L**	**Calculated weight ratio *Rr*ADH to SiO_2 supraparticle**
*Rr*ADH	175.092	0.309	0.176	-	-
SiO_2-*Rr*ADH supraparticles	20 120.522	0.466	0.0023	264.77	1 : 76

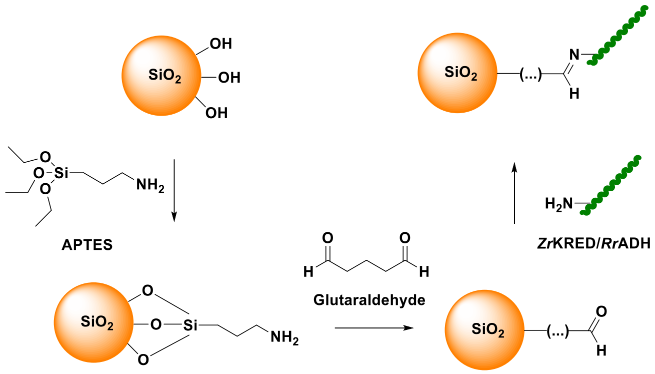

Figure S 7. Glutaraldehyde-functionalization of silica particles and subsequent immobilization of *Rr*ADH by imine formation.[46]

Procedure for the NADH depletion study using SiO_2-*Rr*ADH supraparticles and gas chromatography

For determination of NADH depletion, a 1 mL reaction mixture, containing 0.5 mL NADH solution (c (stock) = 10 mM) in MOPS buffer (50 mM, pH 6.8) and 5 mg/mL of *Rr*ADH of SiO2-RrADH supraparticle suspension (c_{stock} (SiO_2-*Rr*ADH) = 133.3 mg/mL, c (*Rr*ADH, estimated) = 1.333 mg/mL) are mixed together. A phenacyl fluoride solution (c = 20.4 mM) was prepared in MOPS buffer (50 mM, pH 6.8), containing 2 v/v% DMSO and added to the reaction solution, together with an appropriate amount of MOPS buffer for reaching 1 mL of total volume. Each minute, a sample (50 mL) was taken and diluted with 1 mL ethyl acetate for quenching the reaction, dried over $MgSO_4$ and analyzed by GC.

Table S 6. Reaction details for the NADH depletion study with SiO_2-*Rr*ADH supraparticles. T = 25 °C.

#	V_{total}	V_{NADH}	$V_{phenacyl\ fluoride}$	$c_{phenacyl\ fluoride}$	$V_{MOPS\ buffer}$	$V_{SiO2\text{-}RrADH}$ (c_{stock} = 133.33 mg/mL)
1	1 mL	0.5 mL	0.1 mL	2.050 mM	0.400 mL	3.75 µL
2	1 mL	0.5 mL	62.5 µL	1.250 mM	0.437 mL	3.75 µL
3	1 mL	0.5 mL	30.0 µL	0.625 mM	0.470 mL	3.75 µL

4	1 mL	0.5 mL	22.5 µL	0.469 mM	0.477 mL	3.75 µL
5	1 mL	0.5 mL	15.0 µL	0.313 mM	0.485 mL	3.75 µL
6	1 mL	0.5 mL	5.0 µL	0.106 mM	0.495 mL	3.75 µL

General Procedure for the reduction of phenacyl fluoride 2 with *Rr*ADH or SiO_2-*Rr*ADH and NAD^+/isopropanol in batch mode

For determination of time-dependent courses of the enantioselective reduction of phenacyl fluoride **2**, 2 mg of phenacyl fluoride (0.014 mmol, 1 eq.) were dissolved in 15 µL isopropanol. Subsequently, an appropriate amount of MOPS buffer (50 mM, pH 6.8) and the respective catalyst were added to reach a final reaction volume of 0.5 mL. The reaction is started by addition of 6.7 µl of a NADH solution in MOPS buffer (c = 7 mM). A 50 µL sample of the respective reaction solution was taken after 15, 30, 60 and 120 min, diluted and quenched by addition of 1 mL ethyl acetate. The samples were dried over $MgSO_4$ prior to GC analysis for yield and ee determination.

Table S 7. Reaction parameters for time-dependent enantioselective reduction of phenacyl fluoride **2** (c_0 = 30 mM), catalyzed by *Rr*ADH or SiO_2-*Rr*ADH supraparticles. T = 25 °C.

#	V_{total}	$V_{Isopropanol}$	V_{NAD^+}	$V_{MOPS\ buffer}$	Catalyst	$c_{catalyst,stock}$	$V_{catalyst}$	$c_{catalyst,\ RrADH}$	Yield (**3**) t = 30 min
1	0.5 mL	15 µL	6.7 µL	157 µL	*Rr*ADH	0.187 mg/mL	321 µL	120 µg/mL	100%
2	0.5 mL	15 µL	6.7 µL	318 µL	*Rr*ADH	0.187 mg/mL	160 µL	60 µg/mL	95%
3	0.5 mL	15 µL	6.7 µL	425 µL	*Rr*ADH	0.187 mg/mL	54 µL	20 µg/mL	60%
4	0.5 mL	15 µL	6.7 µL	452 µL	*Rr*ADH	0.187 mg/mL	27 µL	10 µg/mL	35%

5	0.5 mL	15 µL	6.7 µL	465 µL	*Rr*ADH	0.187 mg/mL	14 µL	5 µg/mL	14%
6	0.5 mL	15 µL	6.7 µL	428 µL	SiO_2-*Rr*ADH supraparticles	133.33 mg/mL	60 µL	120 µg/mL	93%
7	0.5 mL	15 µL	6.7 µL	453 µL	SiO_2-*Rr*ADH supraparticles	133.33 mg/mL	30 µL	60 µg/mL	95%
8	0.5 mL	15 µL	6.7 µL	463 µL	SiO_2-*Rr*ADH supraparticles	133.33 mg/mL	10 µL	20 µg/mL	90%
9	0.5 mL	15 µL	6.7 µL	468 µL	SiO_2-*Rr*ADH supraparticles	133.33 mg/mL	5 µL	10 µg/mL	53%

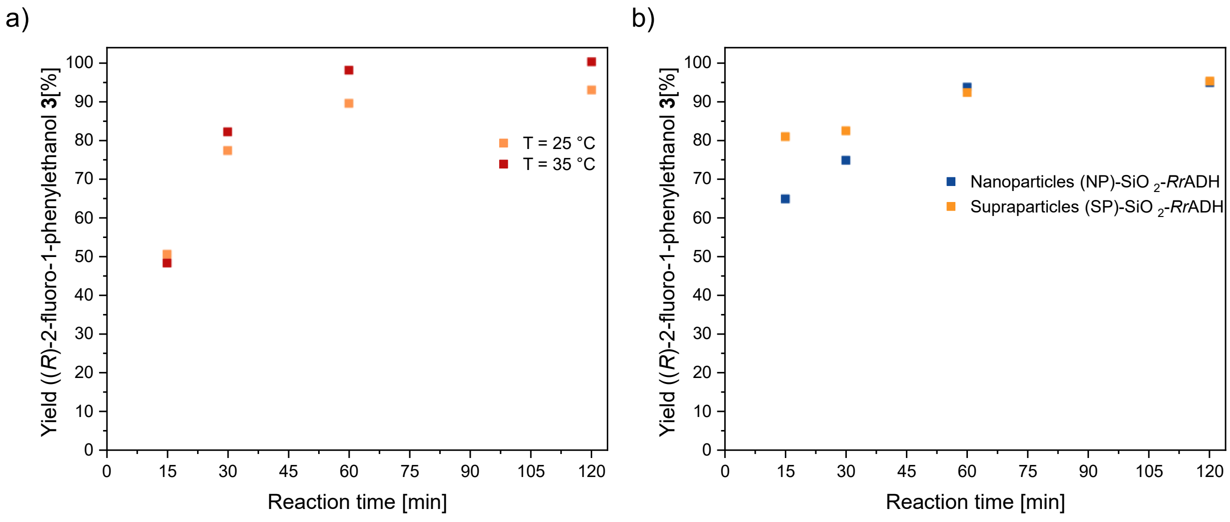

Figure S 8. a) Time-dependent course of (*R*)-2-fluoro-1-phenylethanol **3** yield using a reaction temperature of 25 °C or 35 °C. c_{RrADH} (SiO_2-*Rr*ADH supraparticles) = 20 µg/mL. c (phenacyl fluoride **2**) = 30 mM. B) Time-dependent course of (*R*)-2-fluoro-1-phenylethanol **3** yield using SiO_2-*Rr*ADH nanoparticles or supraparticles. c_{RrADH} (SiO_2-*Rr*ADH nanoparticles/supraparticles) = 20 µg/mL. c (phenacyl fluoride **2**) = 30 mM.

Procedure for the recyclability study using SiO_2-*Rr*ADH supraparticles

For studying the recyclability of SiO_2-*Rr*ADH supraparticles for the enantioselective reduction of phenacyl fluoride **2**, a reaction solution, containing phenacyl fluoride (30 mM), isopropanol (400 mM) and NAD^+ (0.1 mM) in MOPS buffer (50 mM, pH 6.8, 5% methanol) was prepared. 1 mL of the reaction solution was added to 15 µL of SiO_2-*Rr*ADH supraparticles (c_{stock} (SiO_2-*Rr*ADH) = 133.3 mg/mL, c (*Rr*ADH, estimated) = 1.333 mg/mL), reaching a concentration of *Rr*ADH of 20 µg/mL. After a reaction time of 30 min under ambient temperature (T = 25 °C), the reaction suspension was centrifuged by a mini centrifuge. 50 µL of the supernatant were removed for analysis and 1 mL of ethyl acetate was added. The mixture was subsequently dried over $MgSO_4$. Yield and ee values were determined by GC analysis. The centrifuged SiO_2-*Rr*ADH supraparticles were washed three times with MOPS buffer (50 mM, pH 6.8, 5% methanol) and reused for the enantioselective reduction of phenacyl fluoride **2**, repeating the described procedure, until 6 reaction cycles were performed.

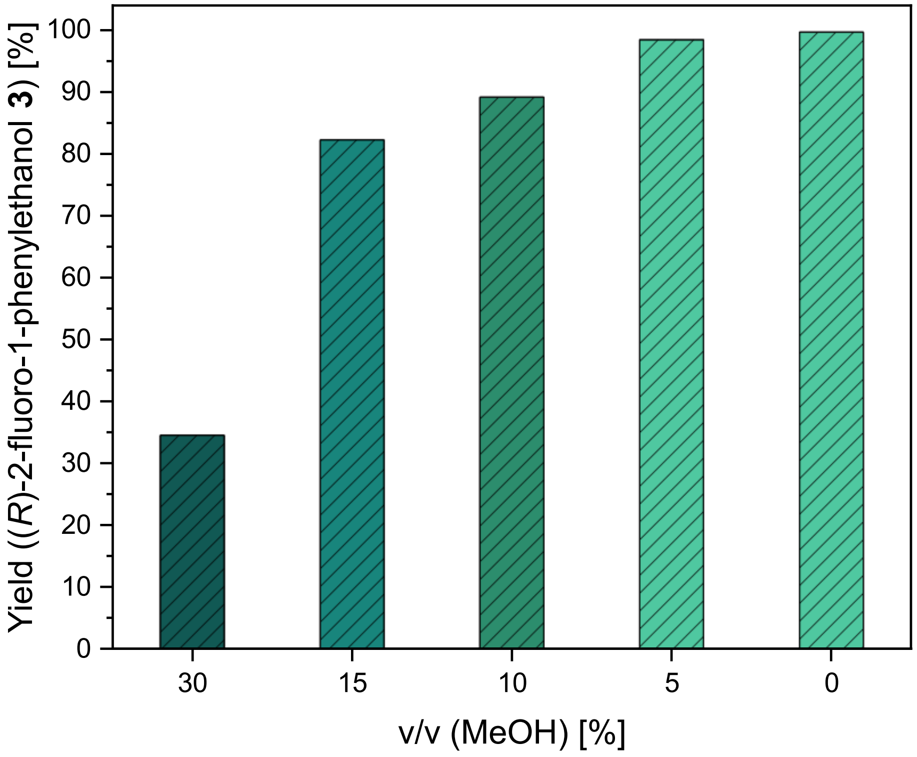

Figure S 9. Yields for the reduction reaction of phenacyl fluoride **2** to (*R*)-2-fluoro-1-phenylethanol **3** with varying concentration of methanol as co-solvent, using SiO_2-*Rr*ADH supraparticles (c (*Rr*ADH) = 20 mg/mL). c_0 (phenacyl fluoride **2**) = 30 mM.

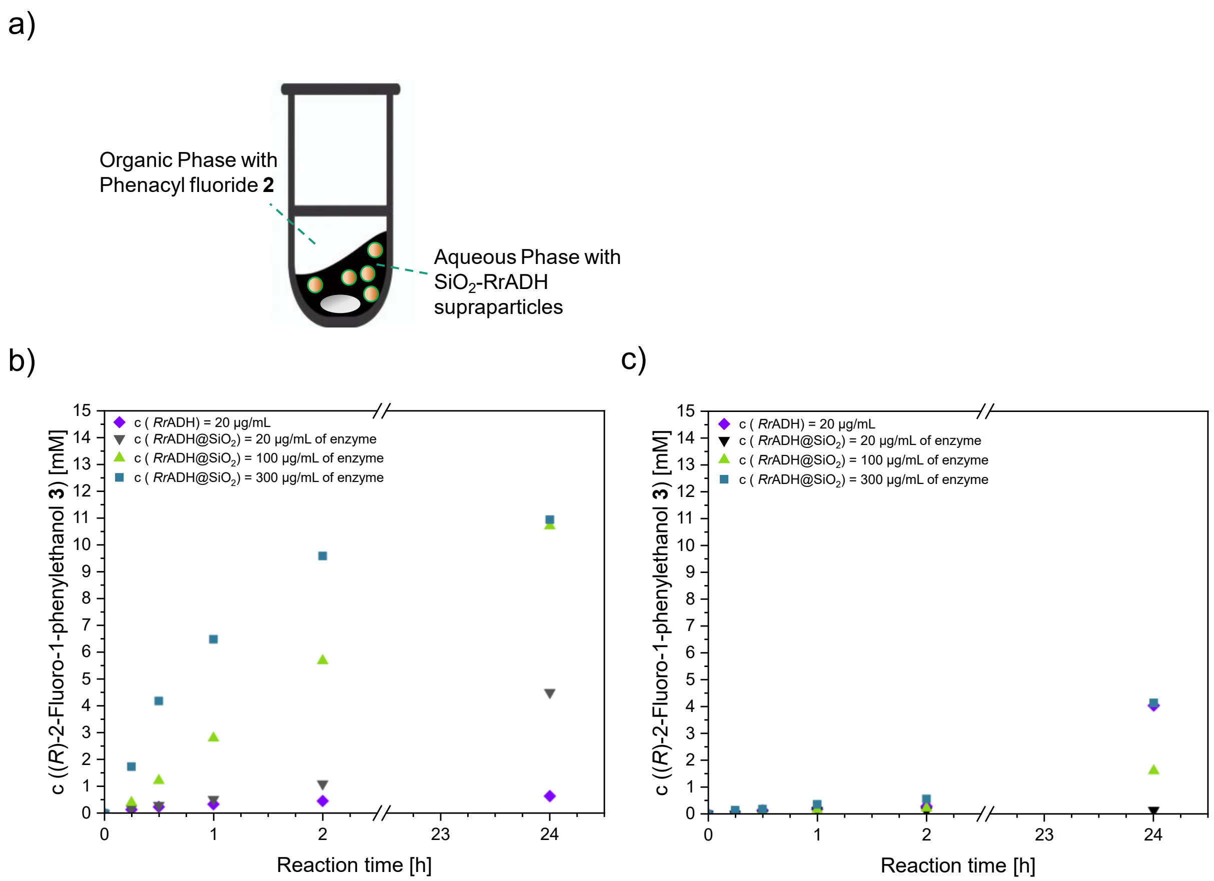

Figure S 10. a) Scheme for a three-phase reaction with SiO_2-*Rr*ADH supraparticles in an aqueous phase and phenacyl fluoride **2** in the organic phase. b, c) Time-dependent course of (*R*)-2-fluoro-1-phenylethanol **3** yield with a three-phase reaction setup, using a 1:1 mixture of a phenacyl fluoride **2** solution in b) cyclohexane or c) ethyl acetate (c (phenacyl fluoride **2**) = 30 mM) and SiO_2-*Rr*ADH supraparticles suspended in MOPS buffer (50 mM, pH 6.8). Different amounts of SiO_2-*Rr*ADH supraparticles were used for the reactions. For GC analysis, samples were directly taken from the organic phase.

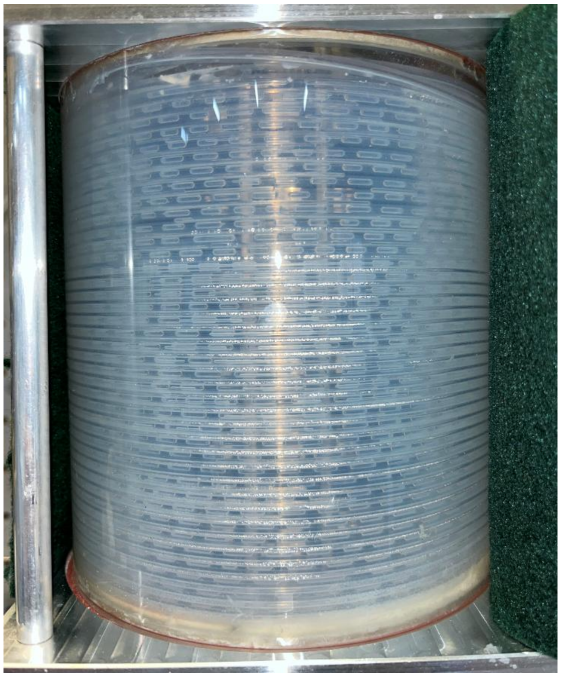

Figure S 11. Capillary reactor R2 during a continuous flow synthesis of (*R*)-2-fluoro-1-phenyl ethanol **3**. The slugs consist of the aqueous catalyst suspension with SiO_2-*Rr*ADH supraparticles and the substrate phenacyl fluoride **2**. Slugs are transported through the reactor and are spatially separated by air slugs.

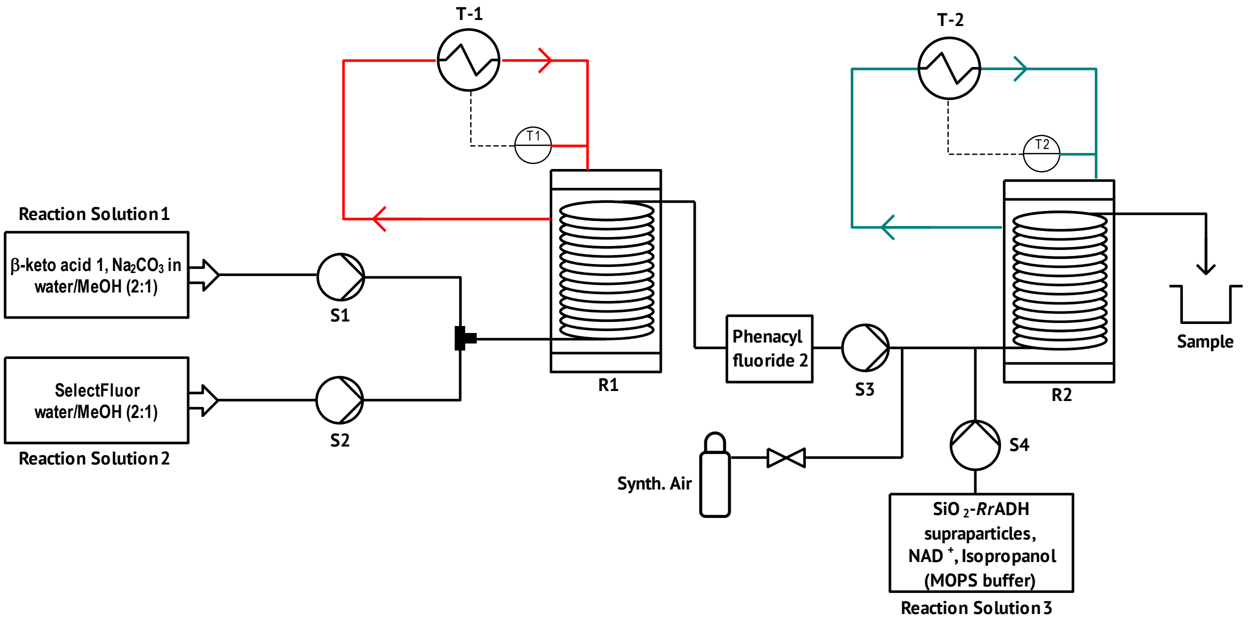

Figure S 12. Detailed flow scheme for the two-step synthesis of (*R*)-2-fluoro-1-phenylethanol **3** by decarboxylative fluorination of b-keto acid **1** (3-oxo-3-phenylpropanoic acid) to the intermediate phenacyl fluoride **2** and subsequent enantioselective reduction, catalyzed by SiO_2-*Rr*ADH supraparticles. Reaction solution 1 and 2 are transported by the syringe pumps S1 and S2 and combined by an interconnected Y-piece. The reaction occurs in the capillary reactor R1, which is tempered by thermostat T-1. Temperature is measured by a thermocouple. The reaction solution is stored in a reservoir and continuously fed into the next part of the lab plant by syringe pump S3. Slug flow is generated by generating a synthetic air flow, precisely

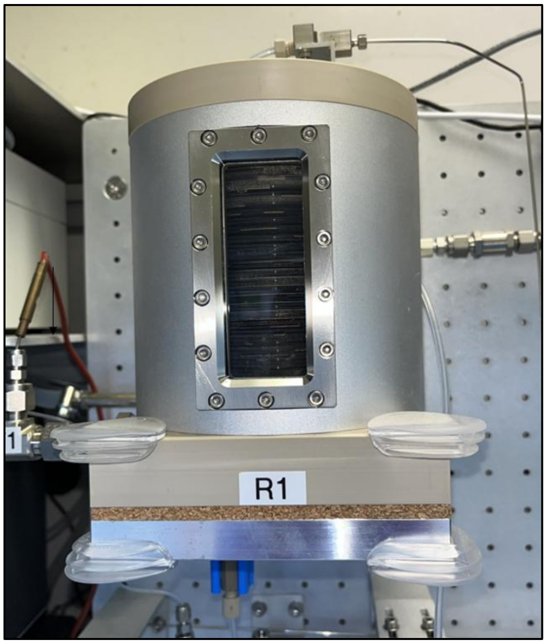

Figure S 13. Thermal capillary reactor (R1) for the decarboxylative fluorination of 3-oxo-3-phenyl propanoic acid **1** in continuous flow mode.

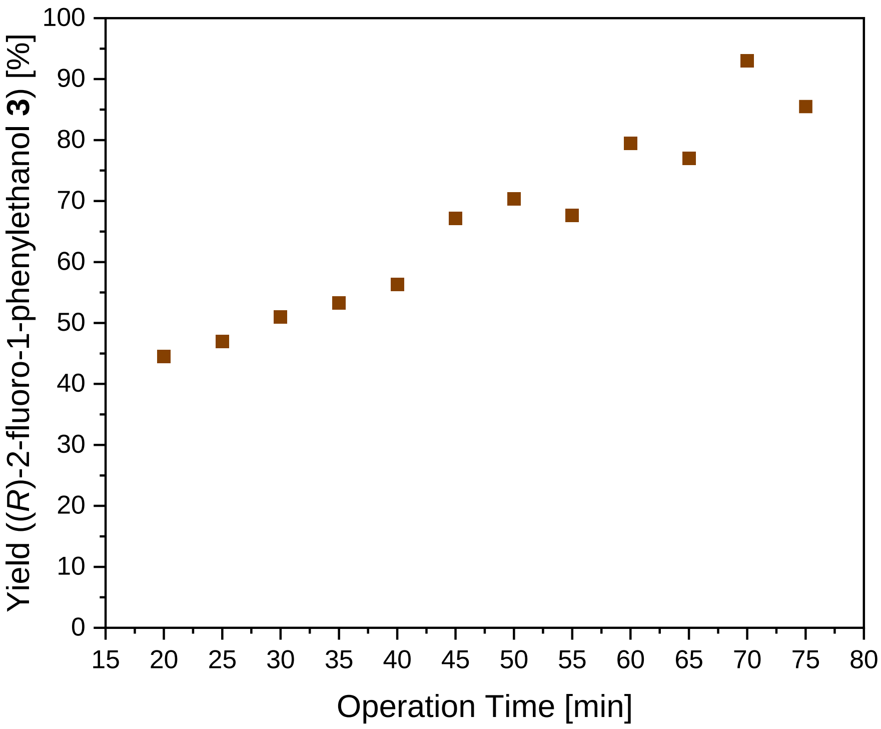

Figure S 14. Yield for the enantioselective reduction of phenacyl fluoride **2** to (*R*)-2-fluoro-1-phenylethanol **3** in continuous flow mode. Samples were taken at intervals of 5 minutes. 50 µL of sample were quenched by addition of ethyl acetate and dried over $MgSO_4$ prior to GC analysis.

3. Experimental Procedures

Synthesis of 3-oxo-3-phenyl propionic acid 1

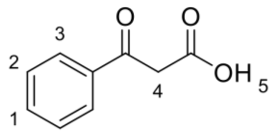

1

2.08 g NaOH (0.052 mol, 1.0 eq.) were dissolved in 50 mL water. 10 g 3-oxo-3-phenylpropanoic ethyl ester (0.052 mol, 1.0 eq.) were added to the solution. The reaction mixture was stirred for 1 day. The reaction mixture is washed with 3x25 mL diethyl ether, the aqueous phase is cooled to 0 °C and acidified with 1M HCl, until a pH value of 1 is reached. The white precipitation is filtrated and washed with water to obtain 3-oxo-3-phenylpropionic acid **1** with quantitative yield, which was used for subsequent reactions without further purification.

R_f (n-H/EE 4:1) = 0.58.

^{1}H-NMR (80 MHz, DMSO-d_6): 8.01-7.52 (m, 5H, H-1, 2, 3), 4.06 (s, 1H, H-4) ppm.

^{13}C-NMR (20 MHz, $CDCl_3$): 198.12, 194.02, 169.48, 137.12, 133.39. 128.91, 128.40, 123.53, 46.14, 26.89 ppm. ^{13}C-NMR data are only obtained from a mixture of **1** and acetophenone due to thermal decarboxylation of **1** during the measurement.

EI-MS: $[M^+]_{calculated}$ = 164.16 m/z. $[M^+]_{measured}$ = 164 m/z.

Synthesis of phenacyl fluoride 2 in continuous flow mode

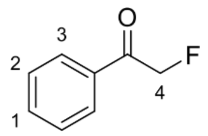

2

The synthesis is based on a previously reported procedure. For a continuous flow experiment, 147.8 mg 3-oxo-3-phenyl propionic acid **1** (0.9 mmol, 1 eq.) are dissolved in 5 mL methanol. Subsequently, 10 mL water and 115.2 mg of Na_2CO_3 (1.1 mmol, 1.2 eq.) are added and the mixture is transferred into a syringe. 382.6 mg SelectFluor® (1.1 mmol, 1.2 eq.) in 15 mL water/MeOH (2:1) are transferred to a second syringe. Both reaction solutions are then mixed by a Y-piece and fed into the capillary reactor with a temperature of 60 °C and a residence time of 60 min. After collection of the reaction solution, it is extracted by 3x30 mL ethyl acetate. The combined organic phase is then washed with 2x20 mL saturated $NaHCO_3$ (aq.) and dried over $MgSO_4$. The organic solvent is removed under reduced pressure. The crude product is purified by column chromatography (Cyclohexane/Ethyl acetate 8:1). The product is obtained with 85% yield.

R_f (Cyclohexane/Ethyl acetate 8:1) = 0.44.

^{1}H-NMR (80 MHz, $CDCl_3$): 7.92 – 7.45 (m, 5H, H-1, 2, 3), 5.51 (d, ^2J = 47 Hz, 2H, H-4) ppm.

^{13}C-NMR (20 MHz, $CDCl_3$): 193.50, 134.20, 129.00, 127,95, 127.82, 88.11, 79.07 ppm.

^{19}F-NMR (75 MHz, $CDCl_3$): -228.67 (t, ^2J = 47 Hz) ppm.

EI-MS: $[M^+]_{calculated}$ = 138.14 m/z. $[M^+]_{measured}$ = 138 m/z.

Synthesis of (*R*)-2-fluoro-1-phenylethanol 3 in continuous flow mode

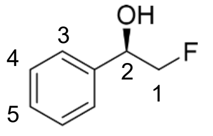

3

For preparation of a continuous flow experiment, 147.6 mg 3-oxo-3-phenyl propionic acid **1** (0.9 mmol, 1 eq.) are dissolved in 5 mL methanol. Subsequently, 10 mL water and 115.2 mg of Na_2CO_3 (1.1 mmol, 1.2 eq.) are added and the mixture (reaction solution 1) is transferred into a 20 mL syringe. 382.2 mg SelectFluor® (1.1 mmol, 1.2 eq.) in 15 mL water/MeOH (2:1) are transferred to a second 20 mL syringe (reaction solution 2). Both reaction solutions are later fed into the synthesis plant by syringe pump S1. Additionally, 20 mL of a reaction suspension in MOPS buffer (50 mM, pH 6.8), containing 600 µL of the SiO_2-*Rr*ADH supraparticle stock (c_{stock} (SiO_2-*Rr*ADH) = 133.3 mg/mL, c (*Rr*ADH, estimated) = 1.333 mg/mL, $c_{RrADH,reaction}$ = 20 µg/mL), 536 µL of a 7 mM NAD+ solution ($c_{NAD^+,reaction}$ = 0.1 mM) and 1.2 mL isopropanol (c = 400 mM) were transferred to a syringe (reaction solution 3) and continuously stirred by an external stirring motor.

The continuous flow production starts by dosing both reaction solution 1 and 2 into the synthesis plant (0.125 mL/min for each reaction solution). Both solutions are then mixed by an interposed Y-piece and fed into the capillary reactor R1 with a temperature of 60 °C and a residence time of 60 min. After collection of the reaction solution in a reservoir, it is fed into the second part of the synthesis plant (0.1 mL/min). For slug flow generation, a gas bottle containing synthetic air and a mass flow controller for setting precise gas flow rates are connected to the system by a three-way valve (PTFE, 1.6 mm bore). The reaction suspension 3 is dosed with 0.1 mL/min and mixed with

the intermediate reaction solution, containing phenacyl fluoride **2**, resulting in a combined flow rate of 0.5 mL/min (0.1 + 0.3 + 0.1 mL/min) and a residence time in the capillary reactor R2 of 60 min. Finally, the reaction suspension is collected and extracted with ethyl acetate, dried over $MgSO_4$ and the organic solvent is removed under reduced pressure. The crude product is purified by column chromatography (cyclohexane/ethyl acetate 8:1). The product is obtained with 84% yield.

R_f (Cyclohexane/Ethyl acetate 8:1) = 0.3.

^{1}H-NMR (400 MHz, $CDCl_3$): 7.39 – 7.31 (m, 5H, H-5, 4, 3), 5.04-4.97 (m, 1H, H-2), 4.58-4.34 (m, 2H, H-1), 2.65 (OH) ppm.

^{13}C-NMR (400 MHz, $CDCl_3$): 138.16, 128.67, 128.44, 126.37, 88.95, 86.32, 77.08, 73.07, 72.87 ppm.

^{19}F-NMR (75 MHz, $CDCl_3$): -217.75, -217.94, -218.38, -218.57, -219.00, -219.20 (dt) ppm.

$^2J_{F\text{-}H\text{–}1}$ = 47.3 Hz, $^3J_{F\text{-}H\text{–}2}$ = 14.2 Hz

EI-MS: $[M^+]_{calculated}$ = 140.16 m/z. $[M^+]_{measured}$ = 140 m/z.

4. 1H-, 13C- and 19F-NMR spectra

3-Oxo-3-phenyl propanoic acid 1

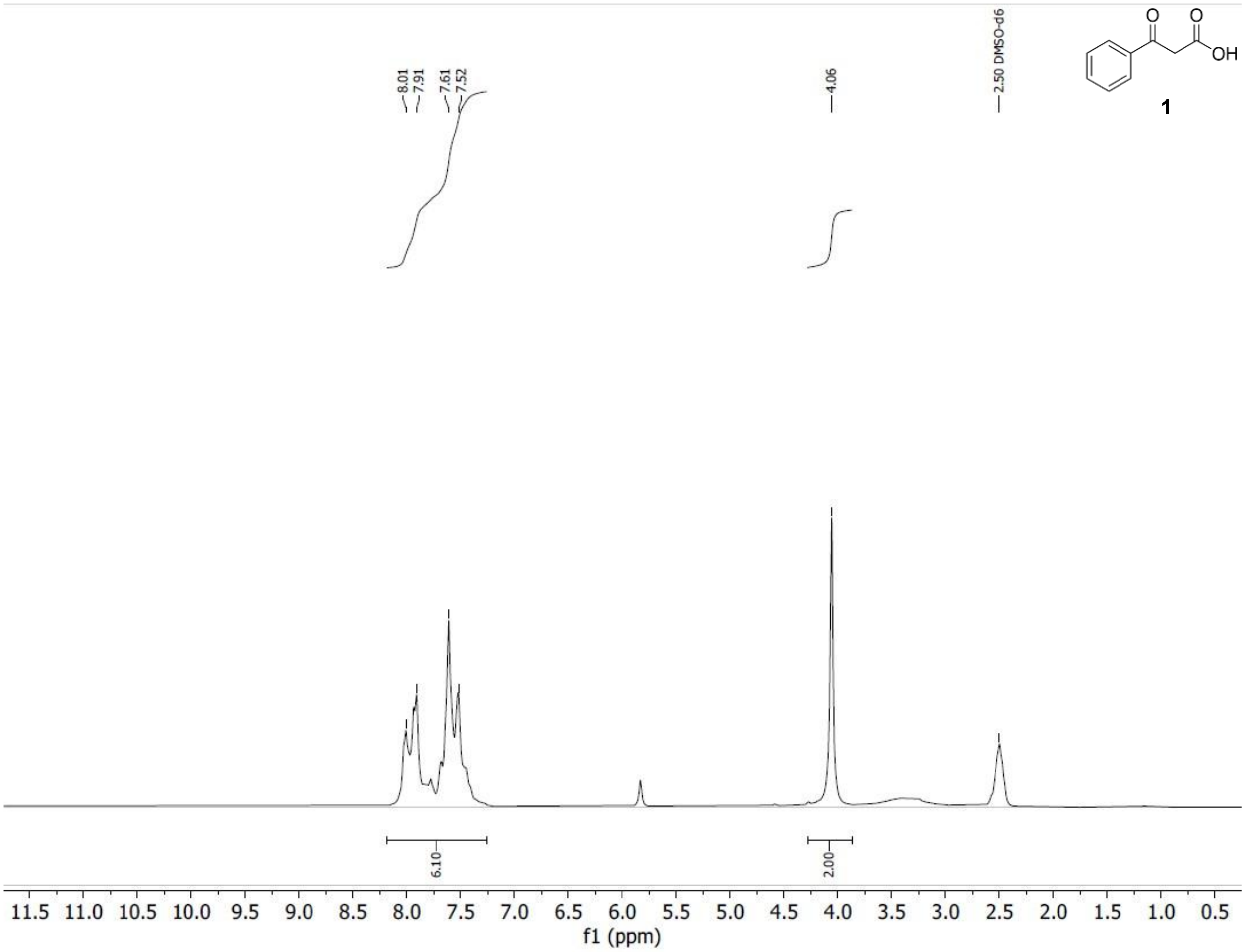

Figure S 15. ^{1}H-NMR spectrum of 3-oxo-3-phenyl propanoic acid. Measured in DMSO-d_6.

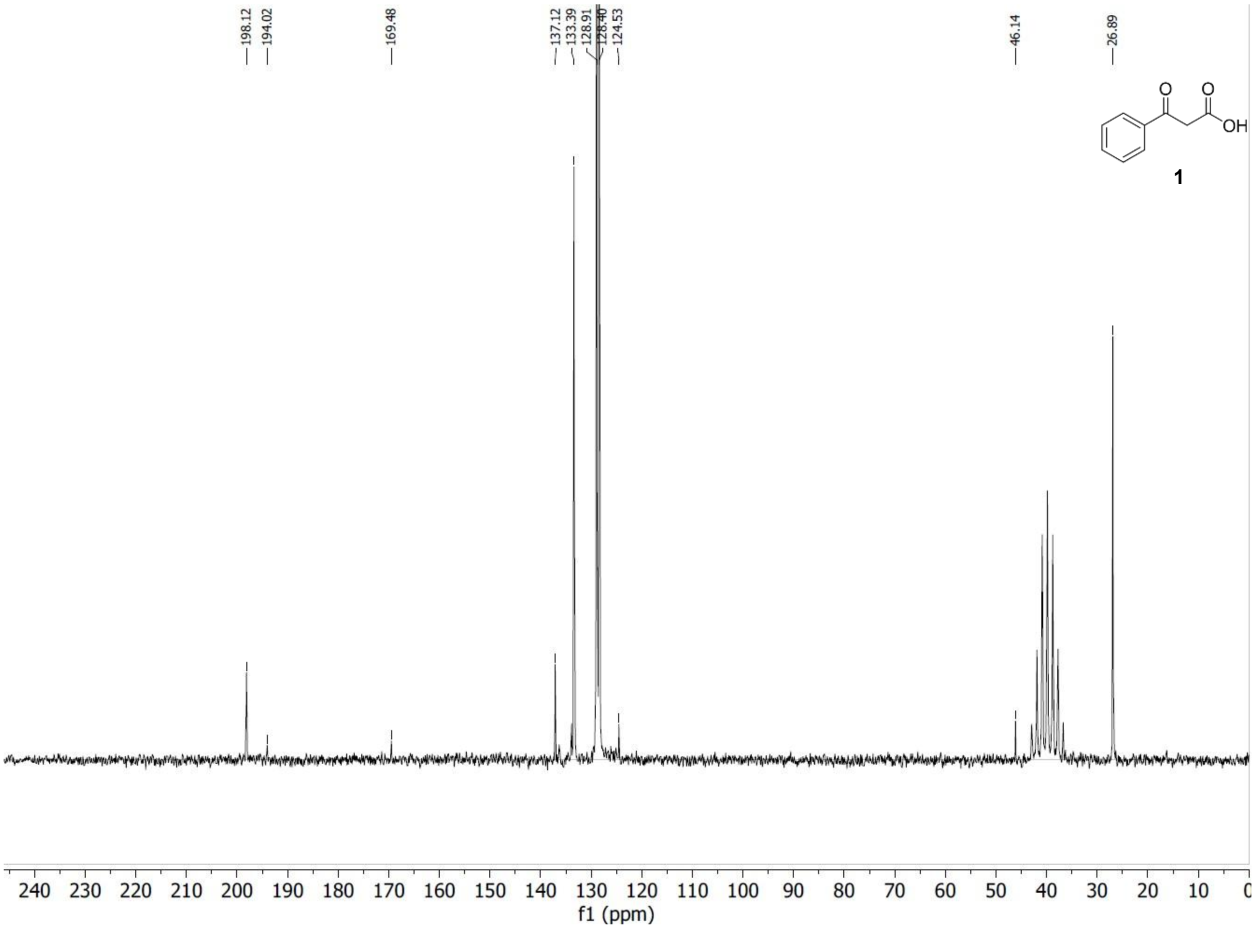

Figure S 16. ^{13}C-NMR of 3-oxo-phenylpropanoic acid **1**. Measured in DMSO-d_6. Due to thermal decarboxylation during the measurement, acetophenone is formed.

Phenacyl fluoride **2**

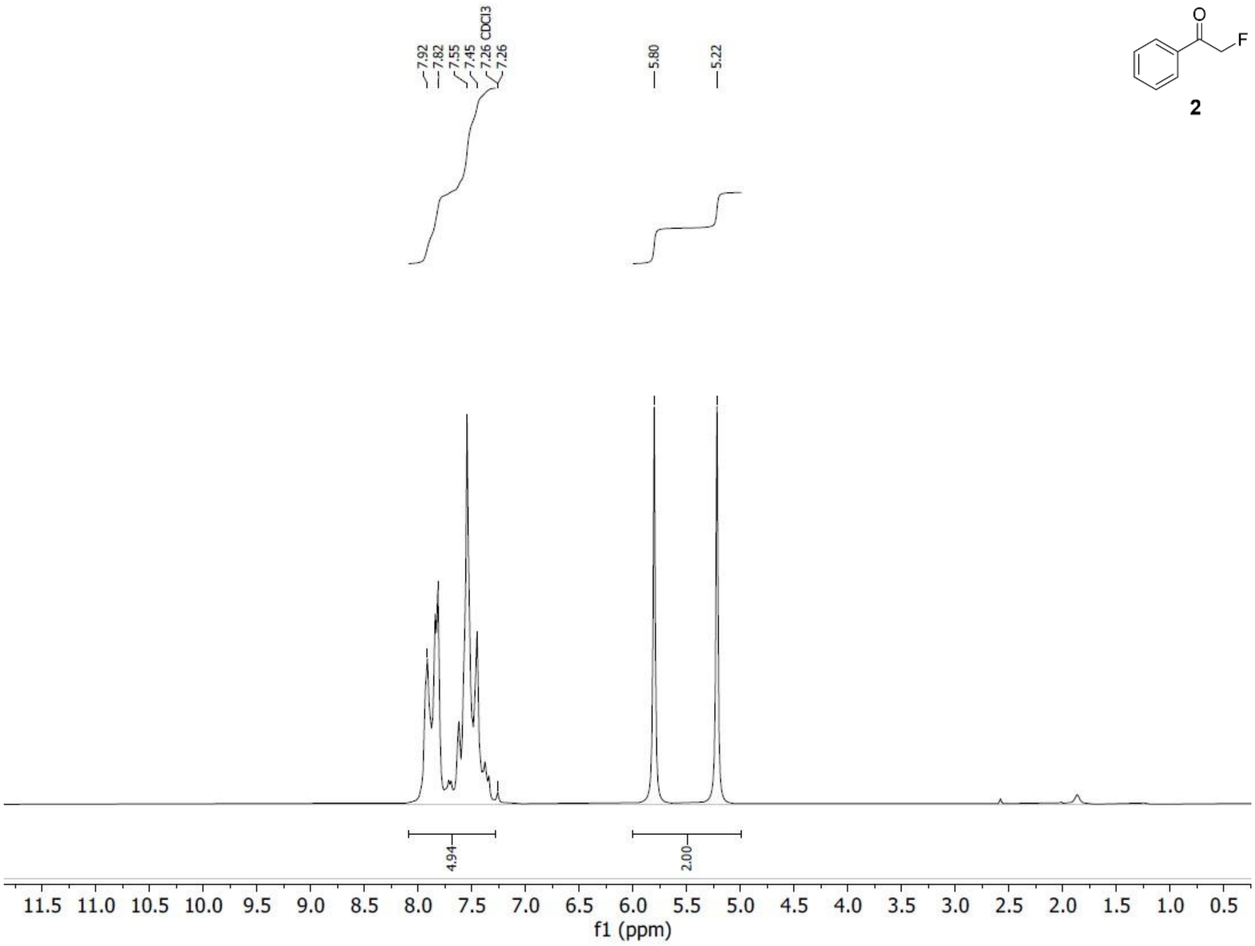

Figure S 17. ^{1}H-NMR spectrum of phenacyl fluoride **2**. Measured in $CDCl_3$.

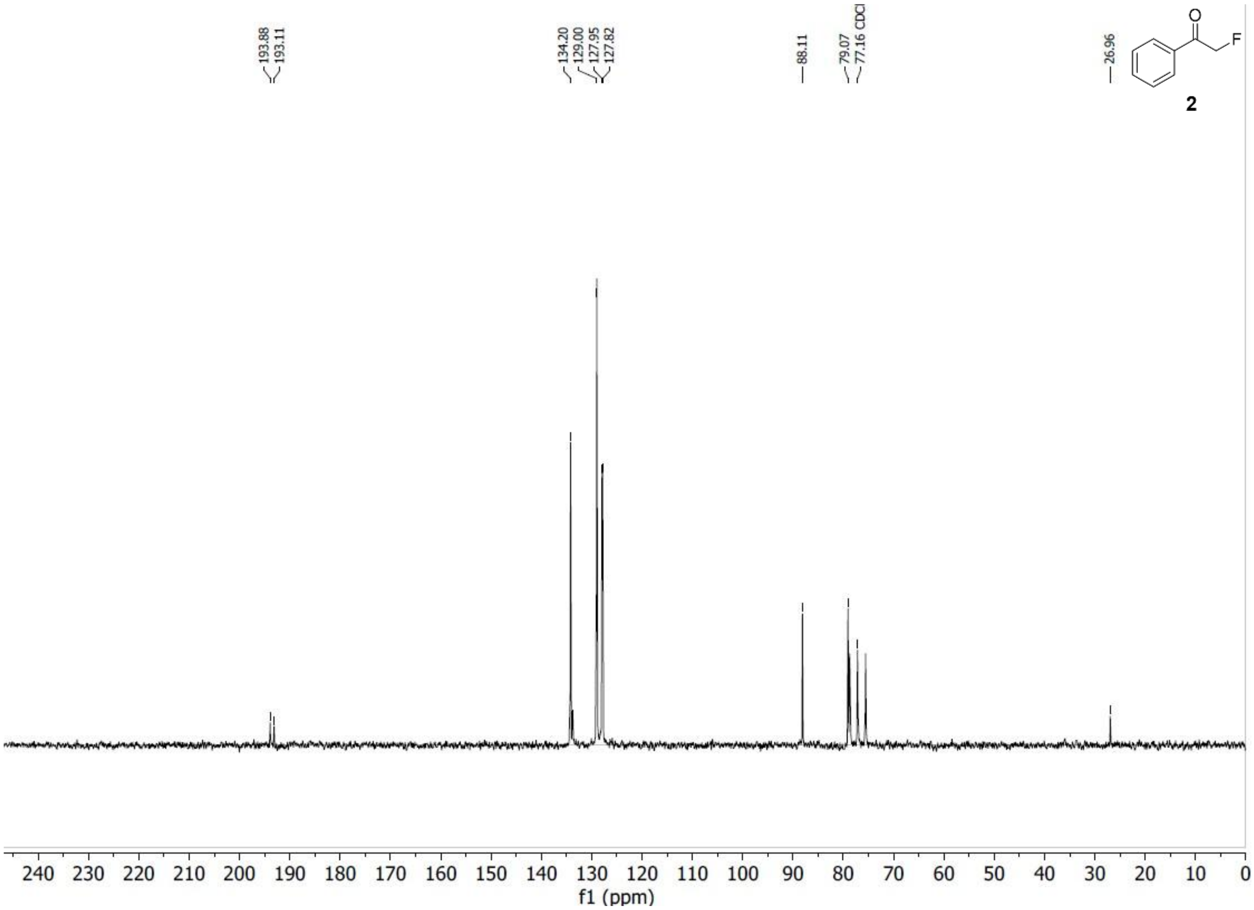

Figure S 18. ^{13}C-NMR of phenacyl fluoride **2**. Measured in $CDCl_3$.

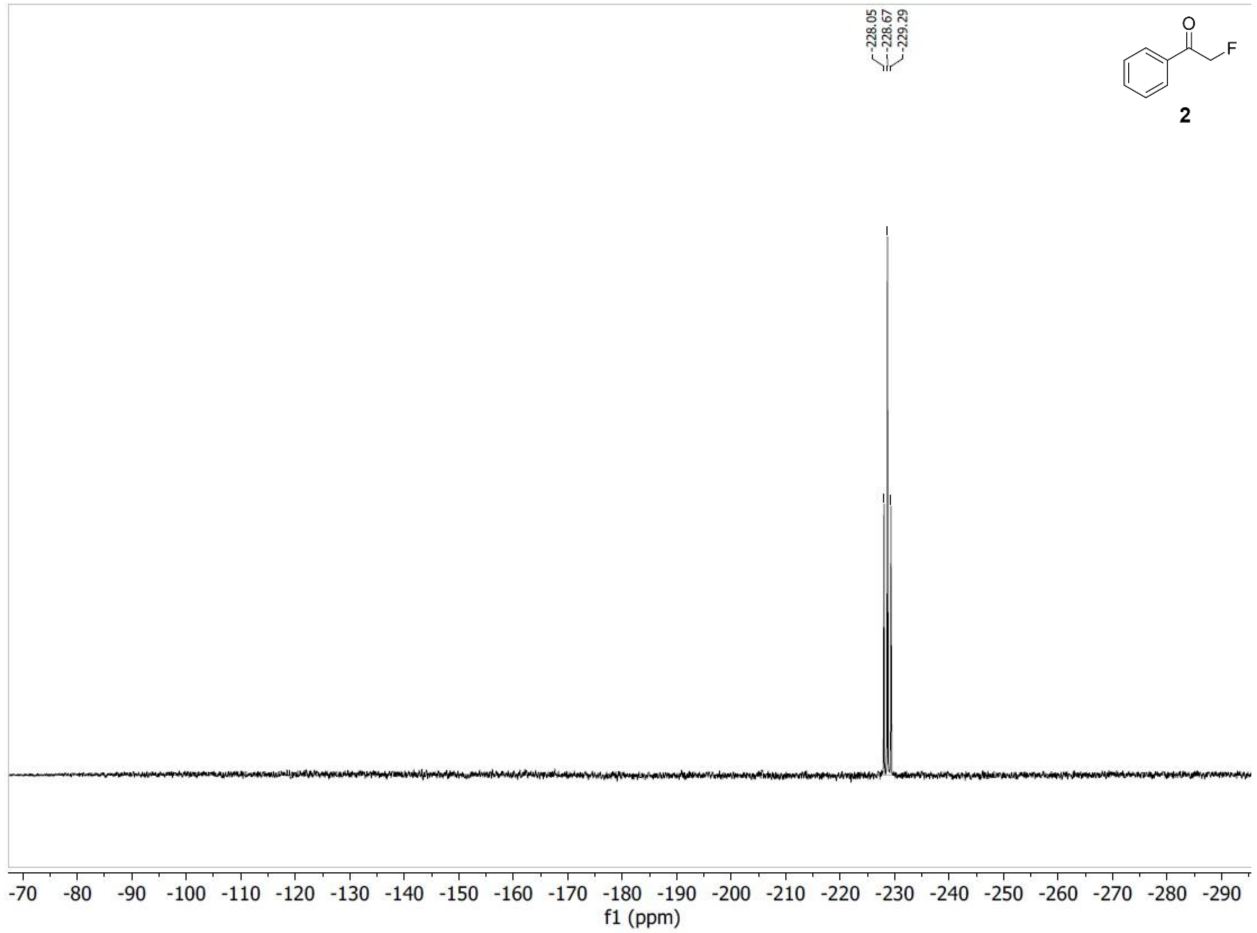

Figure S 19. ^{19}F-NMR spectrum of phenacyl fluoride **2**. Measured in $CDCl_3$.

(*R*)-2-fluoro-1-phenylethanol **3**

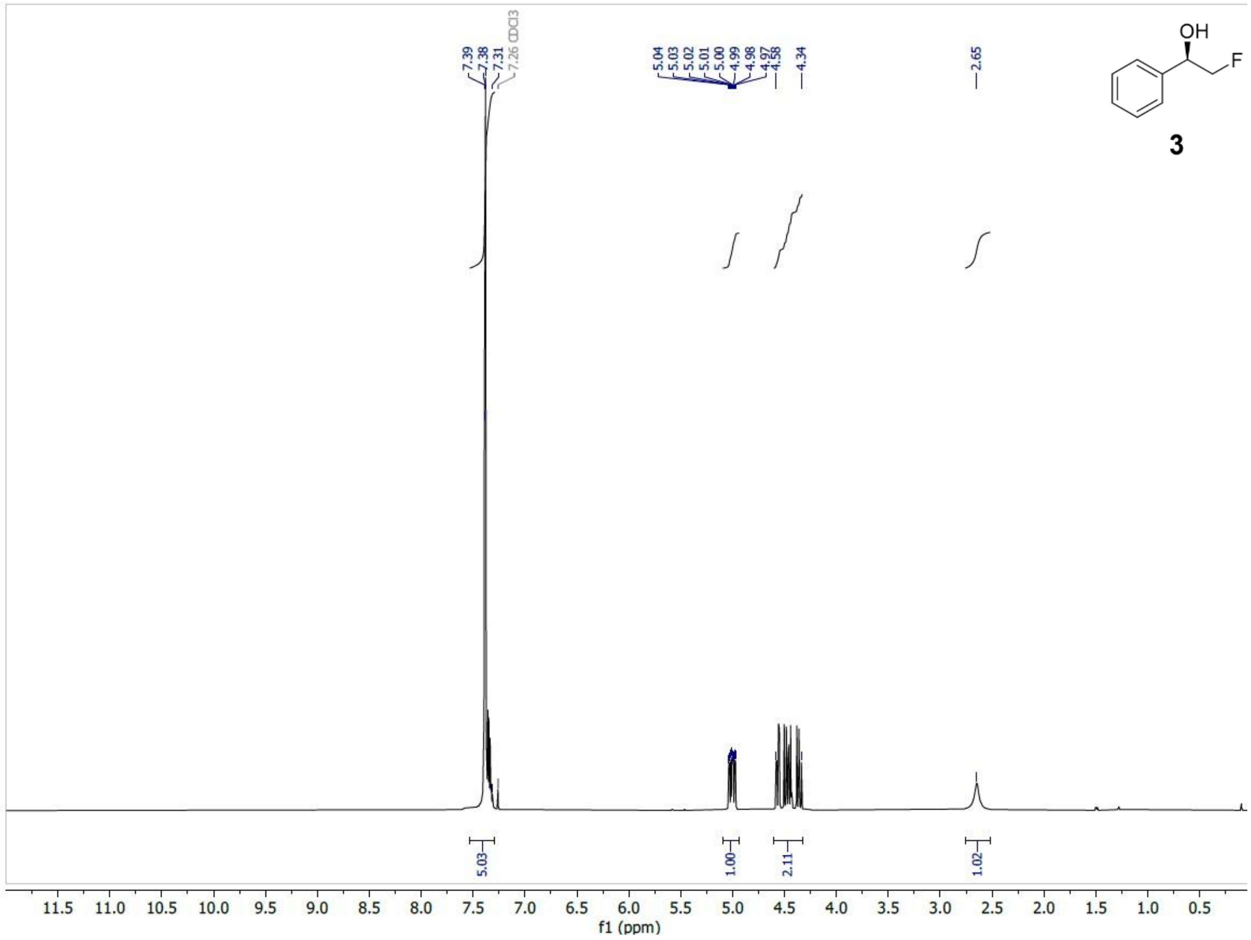

Figure S 20. ^{1}H-NMR of (*R*)-2-fluoro-1-phenylethanol **3**. Measured in $CDCl_3$.

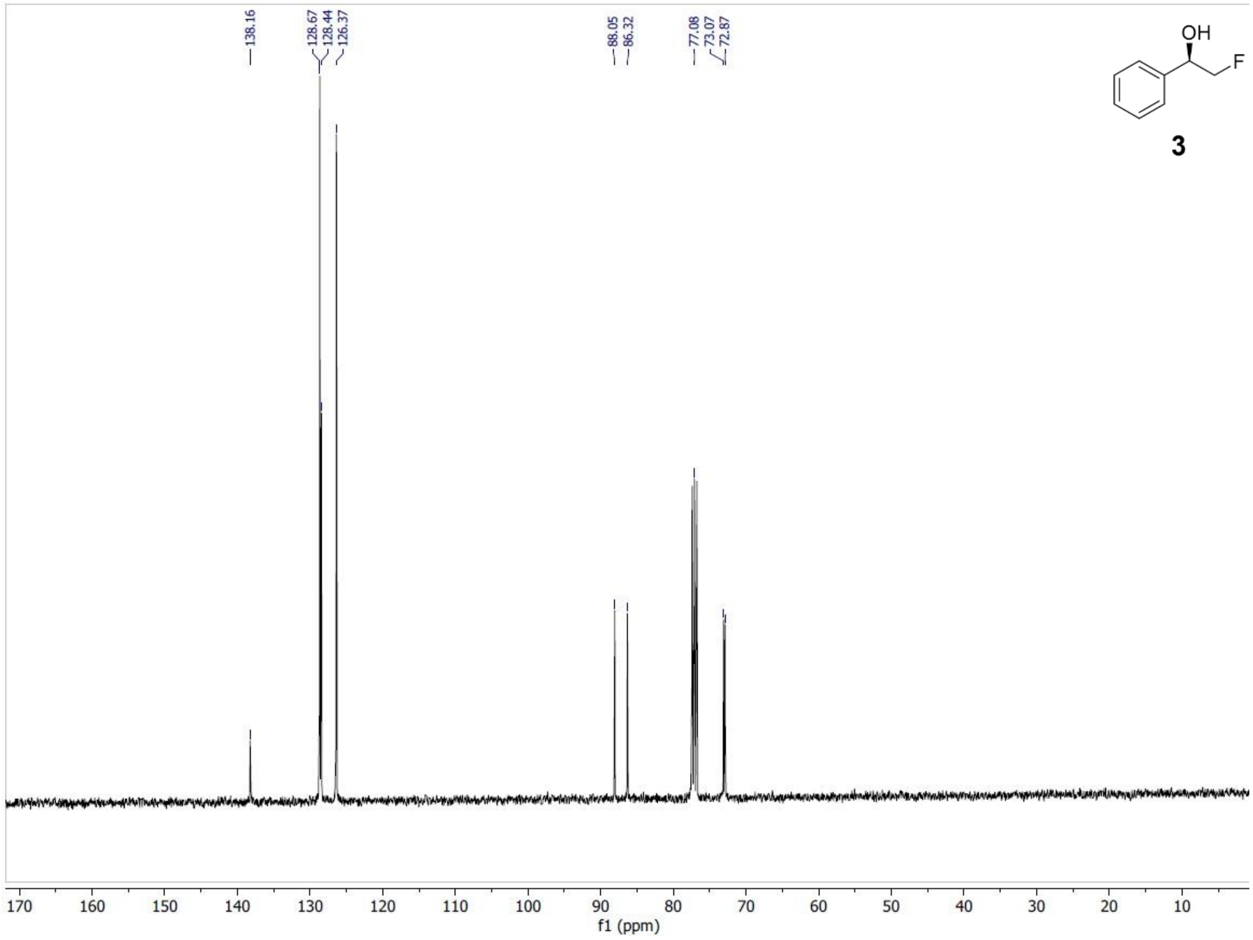

Figure S 21. ^{13}C-NMR of (*R*)-2-fluoro-1-phenylethanol **3**. Measured in $CDCl_3$.

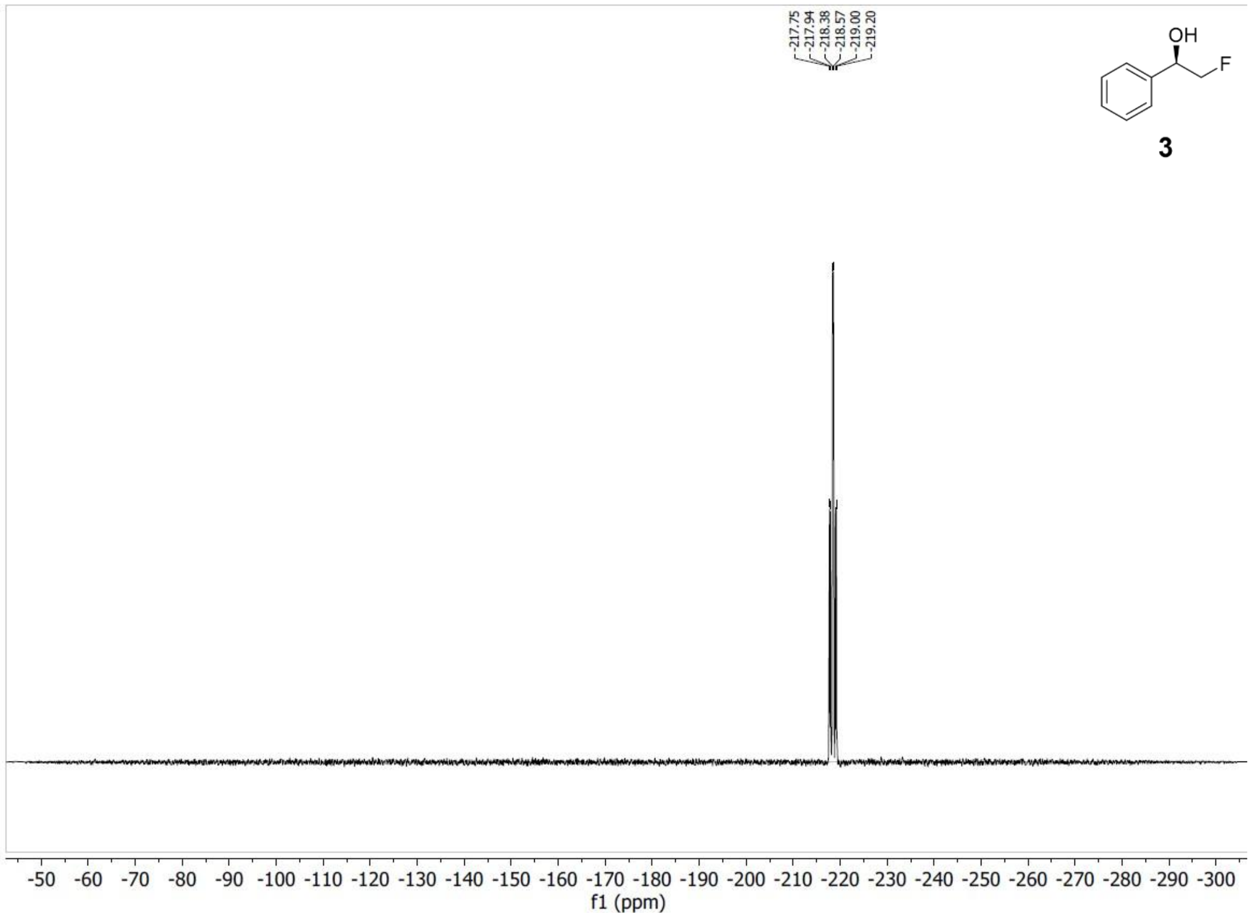

Figure S 22. ^{19}F-NMR of (*R*)-2-fluoro-1-phenylethanol **3**. Measured in $CDCl_3$.

5. GC-MS spectra

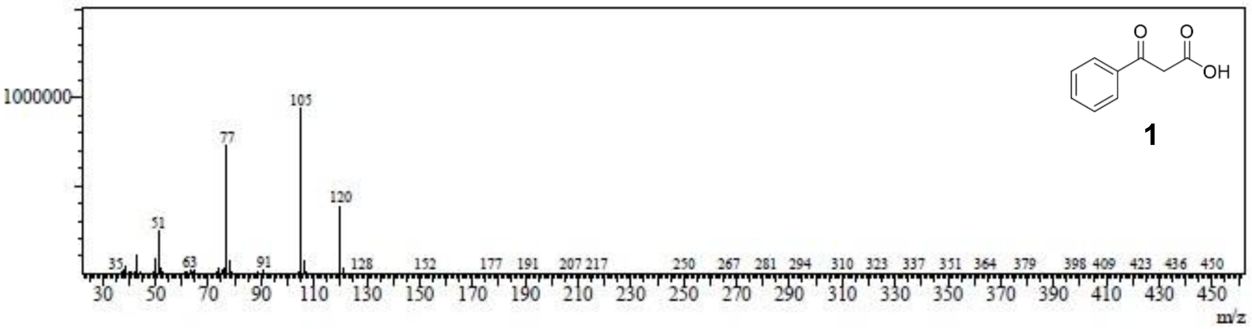

Figure S 23. EI-MS spectrum of 3-Oxo-3-phenylpropanoic acid **1**.

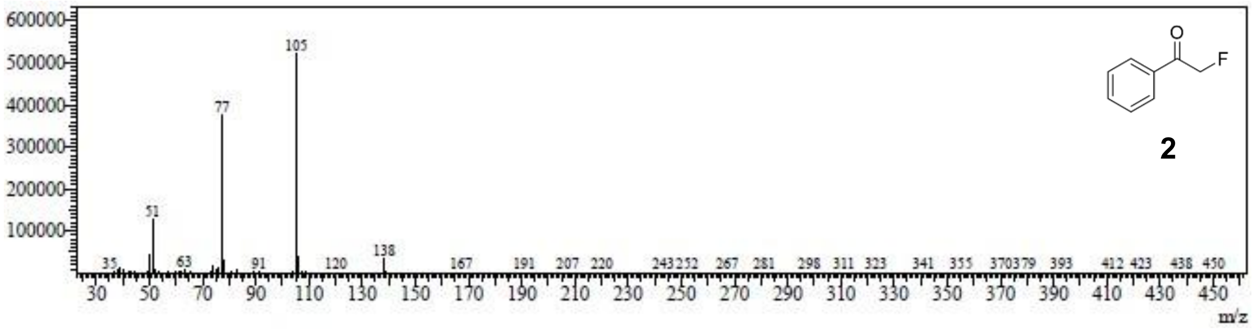

Figure S 24. EI-MS spectrum of Phenacyl fluoride **2**.

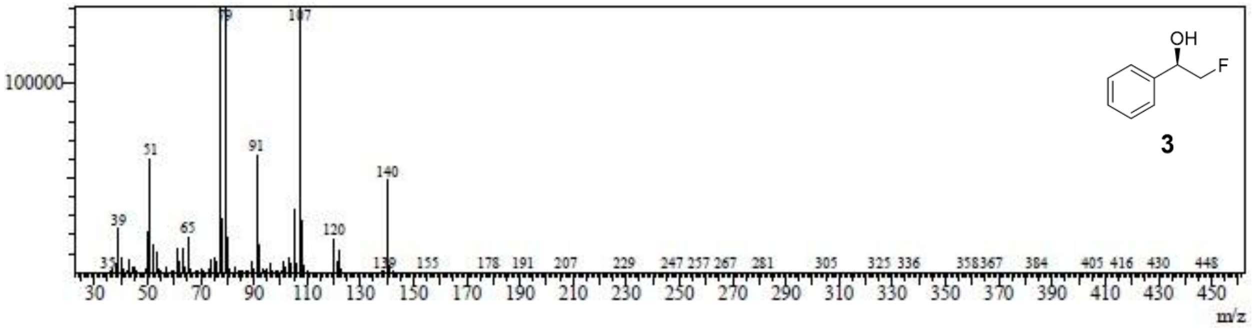

Figure S 25. EI-MS spectrum of (*R*)-2-fluoro-1-phenylethanol **3**.

Literature

[46] B. Herbig, E. Cermjani, D. Hanselmann, et al., *Adv. Funct. Mater.* 2025, e13695.
